# Preterm birth disrupts nephron progenitor cell dynamics predisposing to renal disease

**DOI:** 10.1016/j.isci.2026.117482

**Published:** 2026-09-08

**Authors:** Athar Amleh, Mehran Piran, Julie L.M. Moreau, Aviad Schnapp, Alexander N. Combes, Oded Volovelsky, Morris Nechama

**Affiliations:** 1 Pediatric Nephrology Unit, Hadassah Medical Center and Faculty of Medicine, Hebrew University of Jerusalem, Jerusalem, Israel; 2 Wohl Institute for Translational Medicine, Hadassah-Hebrew University Medical Center, Jerusalem, Israel; 3 Department of Anatomy and Developmental Biology, and Stem Cells and Development Program, Monash Biomedicine Discovery Institute, Monash University, Melbourne, VIC, Australia

**Keywords:** preterm birth, nephron progenitor cells, kidney development, nephron endowment, chronic kidney disease

## Abstract

Preterm birth is associated with a substantially increased lifetime risk of chronic kidney disease, yet the developmental mechanisms driving this association remain unclear. Using an established mouse model of preterm birth, we demonstrate that premature extrauterine life uncouples nephron progenitor cell (NPC) proliferation from differentiation. Although the nephrogenic window is transiently extended, it fails to rescue nephron endowment. Transcriptomic and biochemical analyses reveal that preterm NPCs mount an immediate stress response, marked by unfolded protein response and endoplasmic reticulum stress signatures. This molecular stress coincides with a disruption of the precise temporal dynamics required for efficient differentiation, predisposing to a permanent nephron deficit. Long-term follow-up confirms that these early defects manifest as adult glomerular hypertrophy, proteinuria, and tubular injury. Our findings identify stress-associated dysregulation of NPC dynamics as a central mechanism linking prematurity to lifelong kidney vulnerability, highlighting the perinatal period as a critical therapeutic window.

## Highlights

• Preterm birth uncouples nephron progenitor proliferation and differentiation
• Extended nephron progenitor retention fails to restore nephron endowment
• Preterm birth reprograms nephron progenitor gene expression
• Kidney developmental defects drive lifelong renal injury and functional decline

## Graphical abstract

**Figure:**
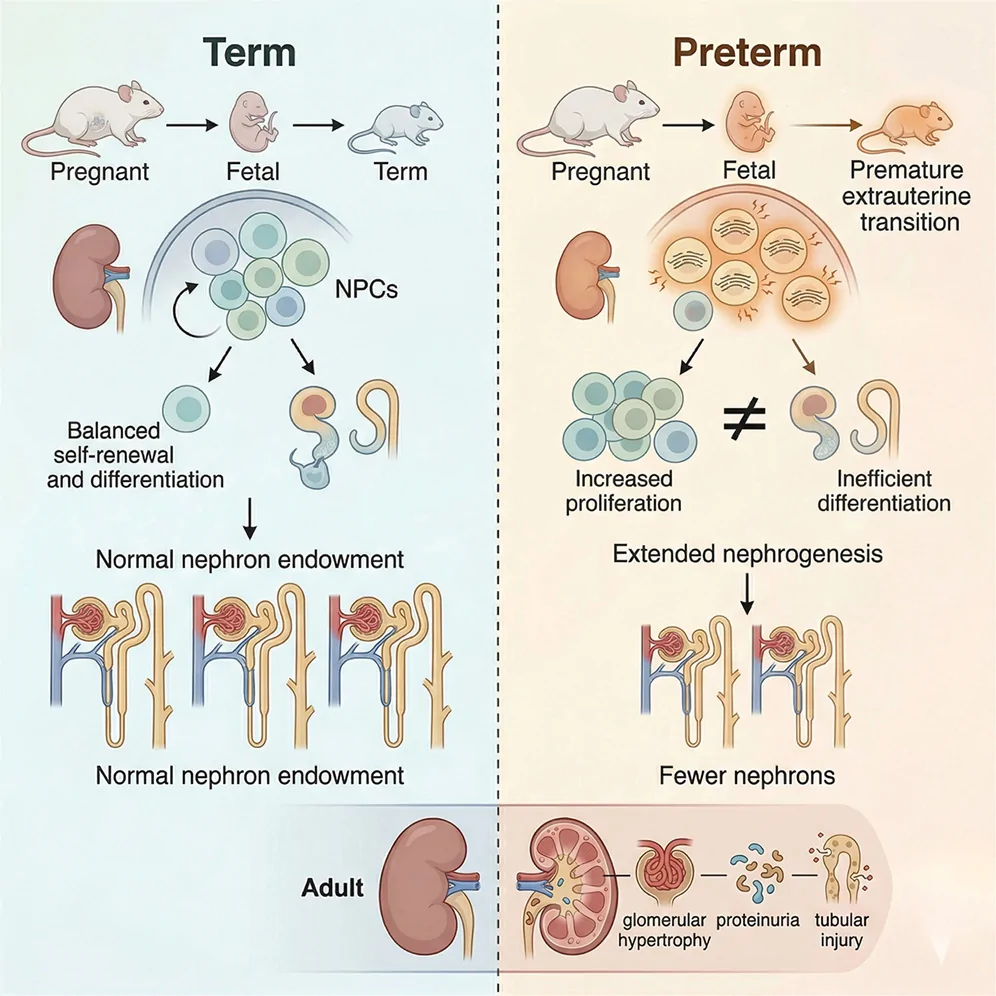

## Teaser

Nephrology; Biological sciences; Developmental biology; Omics

## Introduction

Chronic kidney disease (CKD) constitutes a major global health burden, with an estimated prevalence of 10%–15% and substantial associated morbidity, mortality, and economic costs.^1^^,^^2^ The disease trajectory is increasingly recognized as starting early in development. Accumulating evidence implicates impaired nephron endowment as a critical determinant of susceptibility to renal dysfunction.^3^^,^^4^ Since nephrons are terminally differentiated and non-regenerative, the set established at birth determines the kidney’s functional reserve capacity for life.^4^^,^^5^ This reserve is limited and gradually declines with age and exposure to external insults. As a result, individuals born with fewer nephrons are at a metabolic disadvantage, showing higher risks of hypertension, compensatory glomerular hyperfiltration, and ongoing parenchymal damage.^6^^,^^7^ Therefore, defining the developmental and environmental determinants affecting nephron endowment is essential for understanding susceptibility to kidney disease and represents a critical step toward strategies that mitigate lifelong CKD risk.

Preterm birth, defined as birth occurring before 37 completed weeks of gestation, has emerged as a significant risk factor for diminished nephron endowment.^3^ Worldwide, nearly 10% of infants are born prematurely.^8^ While advances in neonatal intensive care unit (NICU) have markedly increased the survival of infants born at the limits of viability,^9^ this success has revealed a biological conflict. These infants are born while nephrogenesis is still active. Theoretically, the preterm kidney has the potential for continued organogenesis.^10^^,^^11^^,^^12^^,^^13^

However, the abrupt transition to an adverse ex utero environment impairs this process. Instead of completing their developmental program, preterm kidneys frequently exhibit an arrest in nephrogenesis, leading to a permanent structural deficit.^14^^,^^15^^,^^16^ The molecular mechanisms driving this arrest remain incompletely defined. While factors such as oxidative stress, inflammation, and exposure to nephrotoxic medications are known contributors to renal injury in the preterm setting,^17^^,^^18^ it is unclear how these environmental insults are transduced into the nephrogenic niche to alter cell fate. Several rodent models have been developed to study the impact of preterm birth on renal development and long-term outcomes. For instance, surgical cesarean section models in mice have demonstrated that premature delivery can alter nephron progenitor cell (NPC) subpopulations and induce premature NPC differentiation.^19^^,^^20^ Similarly, rat models of neonatal hyperoxia have been utilized to mimic the intensive care environment, revealing significant developmental programming of adult cardio-renal disease.^21^ However, distinguishing the intrinsic developmental impact of the premature extrauterine transition from the confounding systemic inflammatory responses associated with surgical delivery or extreme oxidative stress remains a critical experimental challenge.

Mammalian kidney development requires precise spatiotemporal coordination between NPCs and the branching ureteric bud epithelium.^22^^,^^23^ NPCs constitute a multipotent, self-renewing mesenchymal population that undergoes progressive fate commitment and differentiation to generate functional nephrons.^24^ The balance between NPC self-renewal and differentiation is tightly controlled by complex regulatory networks to ensure proper nephron maturation; premature depletion of NPCs limits final nephron endowment, while prolonged maintenance in the absence of proper differentiation signals can lead to abnormal nephrogenesis.^25^

Previous genetic models, such as those with excess fetal GDNF, have highlighted the plasticity of the nephrogenic program. These models demonstrate that while the postnatal nephrogenic window can be prolonged, it does not necessarily rescue the final nephron endowment if early progenitor dynamics are disrupted.^26^

Determining whether prematurity alters NPC dynamics through changes in proliferation, differentiation, or survival is critical for understanding the mechanisms driving persistent nephron deficits in preterm neonates.

In the present study, using an established mouse model of preterm birth,^27^ we investigate the cellular basis of this defect. We aimed to determine whether the stress of premature extrauterine life triggers an effective uncoupling of NPC proliferation from differentiation, depleting the progenitor pool prematurely, and establishing the structural basis for adult CKD. Our findings define the dysregulated NPC temporal dynamics as a central mechanistic link between prematurity and lifelong kidney vulnerability, highlighting a critical window for intervention.

## Results

### Preterm birth results in permanent loss of nephrons and reduces renal functional reserve

To investigate the sequelae of preterm birth on kidney development, we utilized an established mouse model of prematurity induced by mifepristone, a progesterone antagonist.^27^ To this end, CD1 females were mated, and pregnancy was confirmed by vaginal plug detection, and the morning of vaginal plug detection was designated as postconceptional day 0.5 (PC 0.5). Pregnant females were randomly assigned to one of two groups: (i) a preterm birth group, receiving a single subcutaneous (s.c.) injection of mifepristone (150 μg/kg) at PC17.5 to induce labor at PC18.5 (one day prior to term delivery); and (ii) a term control group injected with the same dose of mifepristone at PC18.5 to induce labor at PC19.5, ensuring that any observed effects are attributable to prematurity rather than drug exposure. Mifepristone administration at PC17.5 consistently triggered preterm birth within 24 h (at PC18.5) without compromising litter size or survival (Figure S1A).

**Figure 1:**
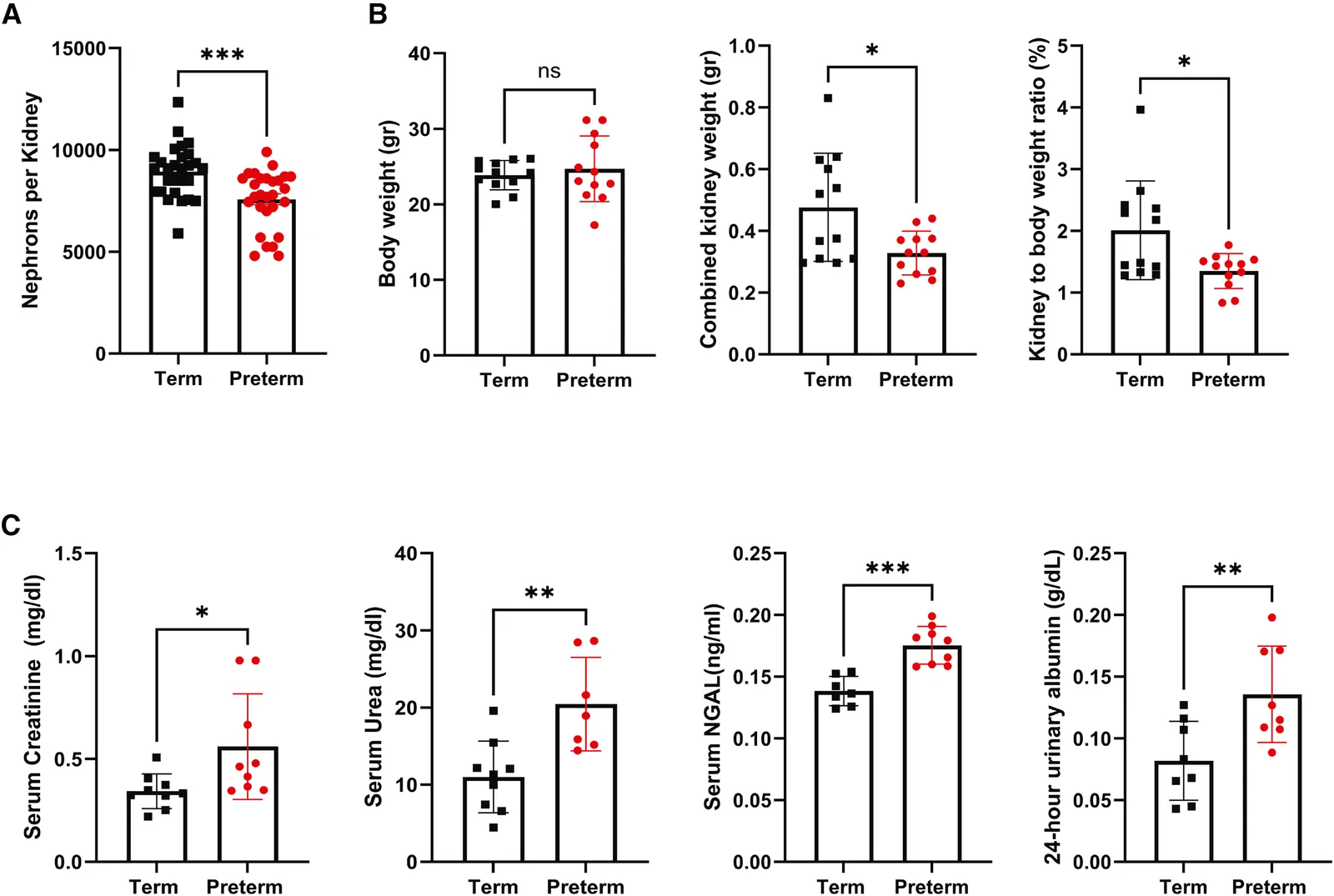
Preterm birth reduces nephron number and impairs renal function (A) Nephron number at P30 in offspring from term and preterm groups, determined by acid maceration. *n* = 20–25 pups per group, three independent litters (biological replicates). (B) Body weight, combined kidney weight and kidney to body weight ratio of offspring as in (A). *n* = 10–15 pups per group, three independent litters (biological replicates). (C) Serum creatinine, urea, NGAL and 24-h urinary albumin concentration levels of offspring as in (A). *n* = 7–9 pups per group, three independent litters (biological replicates). Data are presented as mean ± SEM. A two-tailed Student’s *t* test was used. ∗*p* < 0.05, *∗∗p* < 0.01, *∗∗∗p* < 0.001 versus term. P, postnatal day.

To assess the impact of preterm birth on nephron endowment, we quantified total nephron number in kidneys obtained from term and preterm offspring at postnatal day 30 (P30) as was done before.^28^^,^^29^ Preterm offspring exhibited a significant reduction in nephron number compared with term controls at P30 (Figure 1A). This deficit was accompanied by a lower kidney-to-body weight ratio, driven primarily by decreased kidney mass, rather than differences in somatic growth, as body weight remained comparable between groups (Figure 1B).

We analyzed potential sex-specific effects by examining nephron number, body weight, combined kidney weight, and kidney-to-body weight ratio in male and female mice at P30. These parameters were significantly altered in preterm males but not in females (Figures S1B and S1C). Consequently, all subsequent analyses were performed using males in both groups.

To determine whether reduced nephron number translated into functional impairment, we analyzed serum and urine markers at P30. Preterm males displayed significantly elevated serum creatinine, urea, and neutrophil gelatinase-associated lipocalin (NGAL), alongside increased 24-h urinary albumin concentration, consistent with renal dysfunction secondary to nephron deficit (Figure 1C). Thus, preterm birth is associated with a reduction in nephron endowment and an immediate functional decline, indicative of a reduced renal reserve.

### Prematurity prolongs the nephrogenic window but uncouples NPC proliferation from differentiation

In term-born mice, nephrogenesis typically concludes three days after birth.^30^ Previous reports have demonstrated that genetic interventions can prolong NPC retention within their niche, extend nephrogenesis, and increase nephron endowment.^31^^,^^32^^,^^33^^,^^34^ It remains unclear how preterm birth affects NPC retention or nephrogenesis duration.

**Figure 2:**
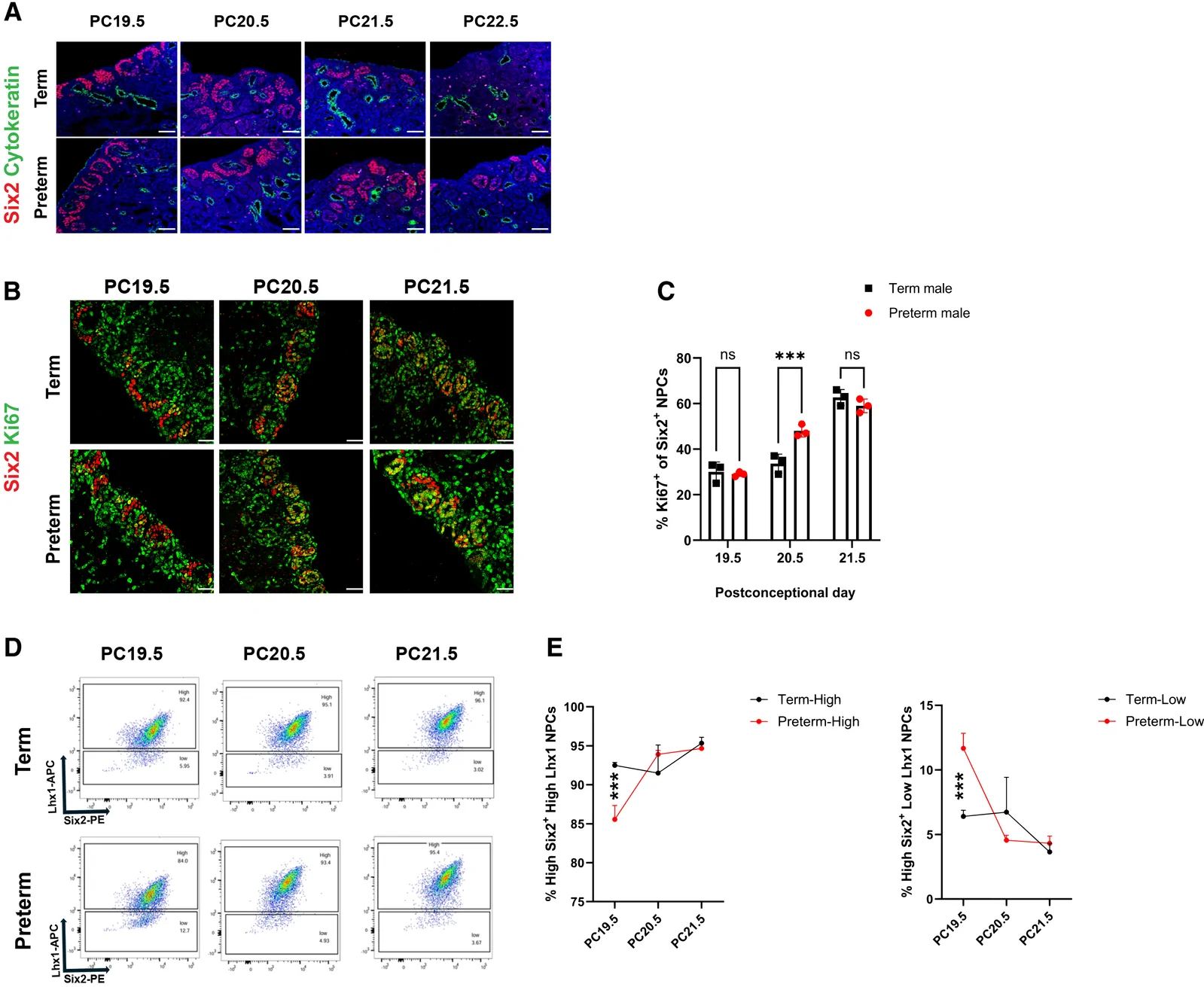
Prematurity extends postnatal nephrogenesis but alters NPC proliferation and differentiation dynamics (A) Representative images of kidney sections from term and preterm offspring at PC19.5 to PC22.5, stained for Six2 (NPC marker), cytokeratin (ureteric bud marker), and DAPI. Scale bars, 30 μm; magnification, ×20. (B) Representative confocal images of kidney sections from male of term and preterm offspring at PC19.5 to PC21.5, stained for Six2 and Ki67 (proliferation marker). Scale bars, 30 μm; magnification, ×20. (C) Quantification of Ki67^+^ proliferating NPCs as in (B), expressed as percentages. *n* = 3 biological replicates per group per postconceptional day (three kidneys from three independent litters). Each dot represents one biological replicate, calculated as the mean of three technical replicates (non-adjacent sections per kidney). (D) Representative FACS analysis of kidney cells from term and preterm offspring at PC19.5 to PC21.5, stained for Six2-PE labeled (NPCs marker) and LHX1-APC labeled (differentiated commitment NPCs marker). (E) Quantification of Six2^high^/LHX1^high^ (left) and Six2^high^/LHX1^low^ (right) NPC populations shown in (D). *n* = 3 biological replicates per group per PC (three independent litters). Data are presented as mean ± SEM. One-way ANOVAs were used. ∗*p* < 0.05, *∗∗p* < 0.01, *∗∗∗p* < 0.001. PC, postconceptional day.

We examined NPC retention in kidneys from PC19.5 to PC22.5 in term and preterm groups, by immunostaining for the NPC marker Six2 and the ureteric bud marker cytokeratin. NPCs were detected until PC21.5 (3 days after birth) in the term group as expected. In preterm mice, Six2^+^ NPCs remained detectable for an additional 24 h (until PC22.5), indicating that prematurity extends NPC retention in the nephrogenic zone (Figure 2A).

To investigate NPC proliferation dynamics, kidney sections (PC19.5–PC21.5) were co-immunostained for Six2 and the proliferation marker Ki67. Confocal analysis revealed a significant increase in Six2^+^Ki67^+^ double-positive NPCs in male preterm pups at PC20.5, suggesting a transient surge in proliferation following preterm birth (Figures 2B and 2C). Notably, this effect was not observed in preterm females at corresponding time points (Figures S2A and S2B), consistent with the sex-specific differences identified in nephron endowment and renal function at P30 (Figure 1). Moreover, this finding was supported by bromodeoxyuridine (BrdU) incorporation assays, which showed a marked increase in Six2^+^BrdU^+^ NPCs at PC20.5 in male preterm pups compared with controls (Figures S2C and S2D). Importantly, this proliferative response was largely confined to the NPC compartment, as no increase in proliferation was observed in proximal tubule cells, as assessed by co-immunostaining for the proximal tubule marker LTL and the proliferation marker Ki67, at earlier time points up to PC20.5. By PC21.5, proximal tubule cells exhibited a decrease in proliferative activity (Figures S2E and S2F), further supporting that the effect was confined to the NPC compartment. TUNEL assays revealed no significant differences in NPC apoptosis between groups, suggesting that cell death is not the primary driver of the observed nephron deficit (Figure S3A).

Increased proliferation suggests enhanced NPC differentiation, as self-renewing NPCs proliferate less than committed progenitors.^35^^,^^36^ To assess whether this proliferative response was coupled with differentiation, single-cell suspensions from PC19.5–PC21.5 kidneys were analyzed for Six2 and LHX1, markers of committed NPCs.^37^^,^^38^ Flow cytometry analysis of NPCs isolated from term and preterm mice kidneys identified two distinct Six2-high subpopulations: undifferentiated NPCs (Six2^high^/LHX1^low^) and differentiated/committed NPCs (Six2^high^/LHX1^high^). At PC19.5, preterm kidneys showed a significant reduction in the committed Six2^high^/LHX1^high^ subpopulation and a relative increase in the undifferentiated Six2^high^/LHX1^low^ pool, indicating impaired differentiation. However, this pattern reversed at PC20.5, where preterm kidneys exhibited an expansion of the Six2^high^/LHX1^high^ population and a reduction in undifferentiated cells (Six2^high^/LHX1^low^), suggesting a compensatory surge in differentiation. By PC21.5, these differences were resolved (Figures 2D and 2E). LHX1 protein expression patterns mirrored these findings, showing lower levels at PC19.5 followed by a significant increase at PC20.5 and PC21.5, and normalization by PC22.5 (Figures S3B and S3C).

Collectively, these findings indicate that preterm birth transiently disrupts NPC differentiation and reduces nephron endowment, despite a compensatory increase in differentiation and prolonged nephrogenesis. This is consistent with reports of continued but often inefficient postnatal nephrogenesis in preterm human neonates and non-human primates.^12^^,^^39^^,^^40^

### Preterm birth is associates with altered NPC gene expression and induces cellular stress at PC19.5

**Figure 3:**
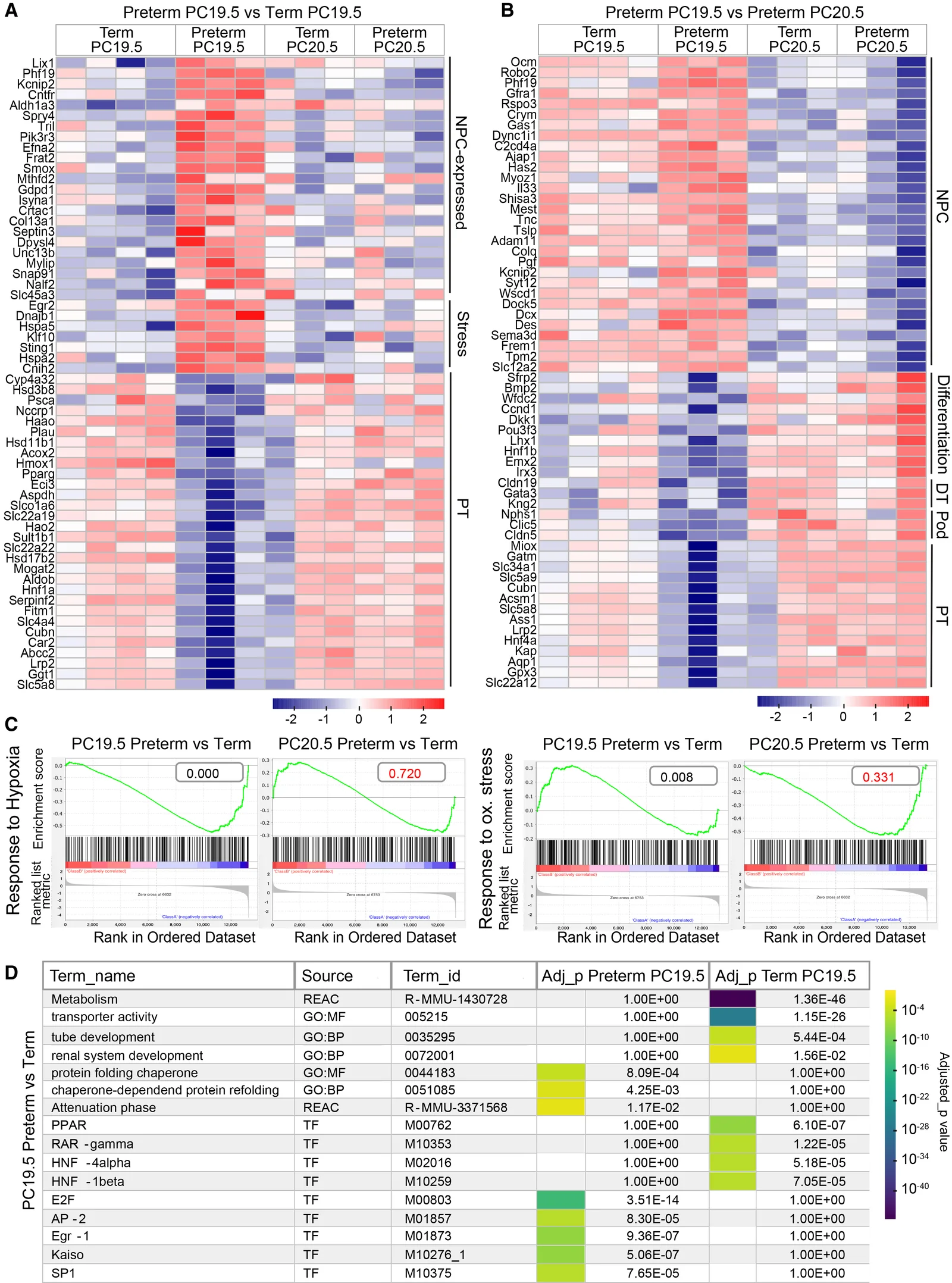
Bulk RNA-seq profiling of Six2^+^ NPCs reveals altered cell stress-associated gene expression in preterm fetal mouse kidneys (A) Heatmap of top differentially expressed (DE) genes between term and preterm PC19.5 NPCs. PT, Proximal tubule; values scaled by row, DE genes: FDR < 0.05, absolute Log fold change (Abs.LogFC) > 0.5. (B) Heatmap of NPC self-renewal and differentiation marker genes differentially expressed between preterm PC19.5 and PC20.5 NPCs. DT, distal tubule; Pod, podocyte; PT, Proximal tubule. Values scaled by row, DE genes: FDR < 0.05, Abs.LogFC > 1. (C) GSEA plots for hypoxia- and oxidative stress-response signatures in preterm versus term NPCs at PC19.5 and PC20.5. Nominal *p* values indicated. The green line shows the enrichment profile, black vertical lines show hits, and the gray histogram shows ranking metric scores. (D) Representative gene set enrichment performed using gProfiler multiquery, which tests signatures separately against genes upregulated in PC19.5 preterm and term NPCs; Adj_p, adjusted *p* value. For each time point, Six2^+^ cells were isolated from kidneys pooled from 3 - 4 independent litters (9–15 pups per pooled sample) to generate a single RNA-seq library. A total of *n* = 3–4 pooled biological replicates per group per PC were sequenced. No technical replicate libraries were performed.

To elucidate the molecular basis of altered NPC dynamics, we analyzed gene expression profiles of NPCs isolated from *Six2 Cre*^*+/tg*^ transgenic mice at PC19.5 and PC20.5 using bulk RNA sequencing (RNA-seq). Differential expression analysis revealed significant transcriptomic shifts between term and preterm NPCs at PC19.5 (Figures 3A and S4A; Data S1). Preterm PC19.5 NPCs showed increased expression of genes enriched in self-renewing NPCs, including *Phf19*, *Cntfr*, *Spry4*, and *Crtac1*,^41^^,^^42^ along with others linked to FGF, retinoic acid, and PI3K signaling pathways involved in NPC regulation (*Spry4*, *Aldh1a3*, and *Pik3r3*).^43^^,^^44^^,^^45^ Genes associated with cell stress and the unfolded protein response (*Dnajb1*, *Hspa2*, *Hspa5*, *Egr2*, *Sting1*, and *Klf10*) were also upregulated in preterm PC19.5 NPCs (Figure 3A). In contrast, genes related to proximal tubule differentiation, including *Pparg*, *Ppara*, *Hnf1a*, *Cubn*, and *Lrp2*, were significantly reduced in PC19.5 preterm NPCs (Figure 3A). Consistent with these findings, custom gene set testing showed enrichment of NPC self-renewal signatures in preterm PC19.5 NPCs (Adjusted *p* value 1.779 × 10^-3^), whereas early proximal tubule signatures were enriched in term PC19.5 (Adjusted *p* value 7.330 × 10^-85^), suggesting that preterm birth suppresses NPC differentiation (Figure S4B).

To determine whether differentiation recovers after preterm birth, we compared preterm NPCs at PC19.5 and PC20.5. Established markers of NPC commitment and early nephron differentiation were increased in preterm PC20.5, while self-renewal markers, including *Robo2*, *Phf19*, *Rspo3*, and *Crym*, were enriched at PC19.5 (Figure 3B; Data S1). No significant differences were detected between term and preterm NPCs at PC20.5, suggesting that the primary molecular disruption occurs during preterm birth.

Gene set enrichment analysis (GSEA) demonstrated significant enrichment of oxidative stress and hypoxic response pathways in preterm NPCs at PC19.5, but not PC20.5 (Figure 3C), with a similar pattern observed for oxidative phosphorylation and mitochondrial electron transport pathways (Figure S4C). These differences may, in part, reflect shifts in proximal tubule signatures and proliferation dynamics between conditions. Directional enrichment analysis across diverse gene set collections supported these findings, revealing protein misfolding and stress-responsive transcriptional programs in preterm NPCs. In contrast, term NPCs showed enrichment of metabolic, kidney development, and transporter activity signatures consistent with ongoing nephron differentiation (Figure 3D).

Importantly, no significant gene expression changes were observed in *Six2 Cre*^*+/tg*^ GFP-negative (non-NPC) kidney cells (Figure S4D), indicating that changes in nephron number and kidney function result from disrupted NPC regulation, rather than effects on other cell types.

We validated and extended these transcriptomic findings by assessing key differentiation and endoplasmic reticulum (ER) stress markers. Preterm NPCs showed a significant upregulation of *Lhx1*, *Wnt4*, and *Wnt9b* expression at PC20.5 by real-time PCR, indicating increased differentiation compared with PC19.5, while no significant changes were observed in the term group (Figure S5A). Consistent with the stress signatures identified by RNA-seq, western blot analysis of whole kidney extracts revealed significantly elevated levels of key ER stress markers-ER chaperone binding immunoglobulin (BiP) and the unfolded protein response sensor protein kinase RNA-like endoplasmic reticulum kinase (PERK) in preterm kidneys from PC20.5 to PC22.5 (Figures S5B and S5C). The transcriptional activation of cell stress pathways at PC19.5 followed by induction of protein effectors, is consistent with previous reports,^46^^,^^47^ suggesting that preterm birth is associated with sustained activation of the unfolded protein response and ER stress pathways shortly after delivery, which strongly correlates with altered nephron progenitor regulation and differentiation dynamics in preterm kidneys.

### Preterm birth induces long-term renal structure and function impairment

**Figure 4:**
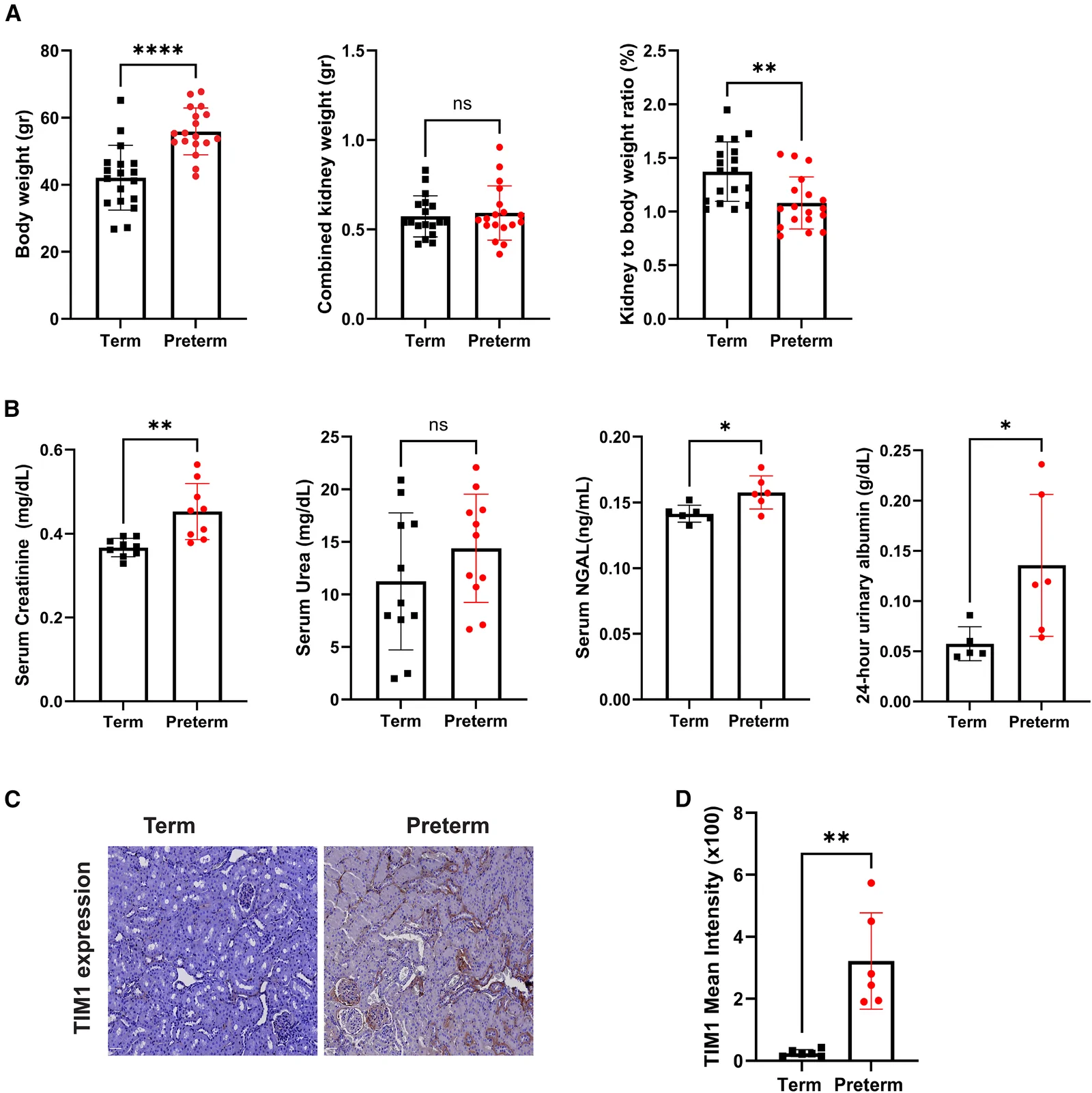
Preterm birth induces long-term renal structure and function impairment (A) Body weight, combined kidney weight and kidney to body weight ratio of offspring at P180. *n* = 15–20 per group, three independent litters (biological replicates). (B) Serum creatinine, urea, NGAL and 24-h urinary albumin concentration levels of offspring as in (A). *n* = 5–11 pups per group, three independent litters (biological replicates). (C) Representative images of TIM1 immunohistochemical kidney staining from mice of the term and preterm groups at P180. Scale bars, 50 μm; magnification, ×20. (D) Quantification of TIM1 expression among term and preterm groups as in (C). *n* = 4–6 biological replicates per group (kidneys from three independent litters). Each dot represents the mean of three technical replicates (non-adjacent sections per kidney). Data are presented as mean ± SEM. A two-tailed Student’s *t* test was used. ∗*p* < 0.05, *∗∗p* < 0.01, *∗∗∗p* < 0.001, ∗∗∗∗*p* < 0.0001 versus term. P, postnatal day.

To assess the long-term consequences of prematurity, we evaluated renal outcomes at 6 months of age (P180). Preterm mice exhibited significantly increased body weight compared with controls, whereas their combined kidney weight remained unchanged, resulting in a significantly lower kidney-to-body weight ratio (Figure 4A).

Biochemically, preterm mice displayed persistent renal dysfunction, characterized by elevated serum creatinine and NGAL levels, as well as significant albuminuria (Figure 4B). Immunohistochemical analysis revealed increased expression of T cell immunoglobulin and mucin domain-containing protein 1 (TIM1), a sensitive marker of tubular injury,^48^ in the kidneys of preterm mice compared with controls (Figures 4C and 4D). These data indicate that early developmental insults lead to permanent functional decline and chronic tubular injury.

### Preterm birth induces long-term glomerular hypertrophy and structural abnormalities

**Figure 5:**
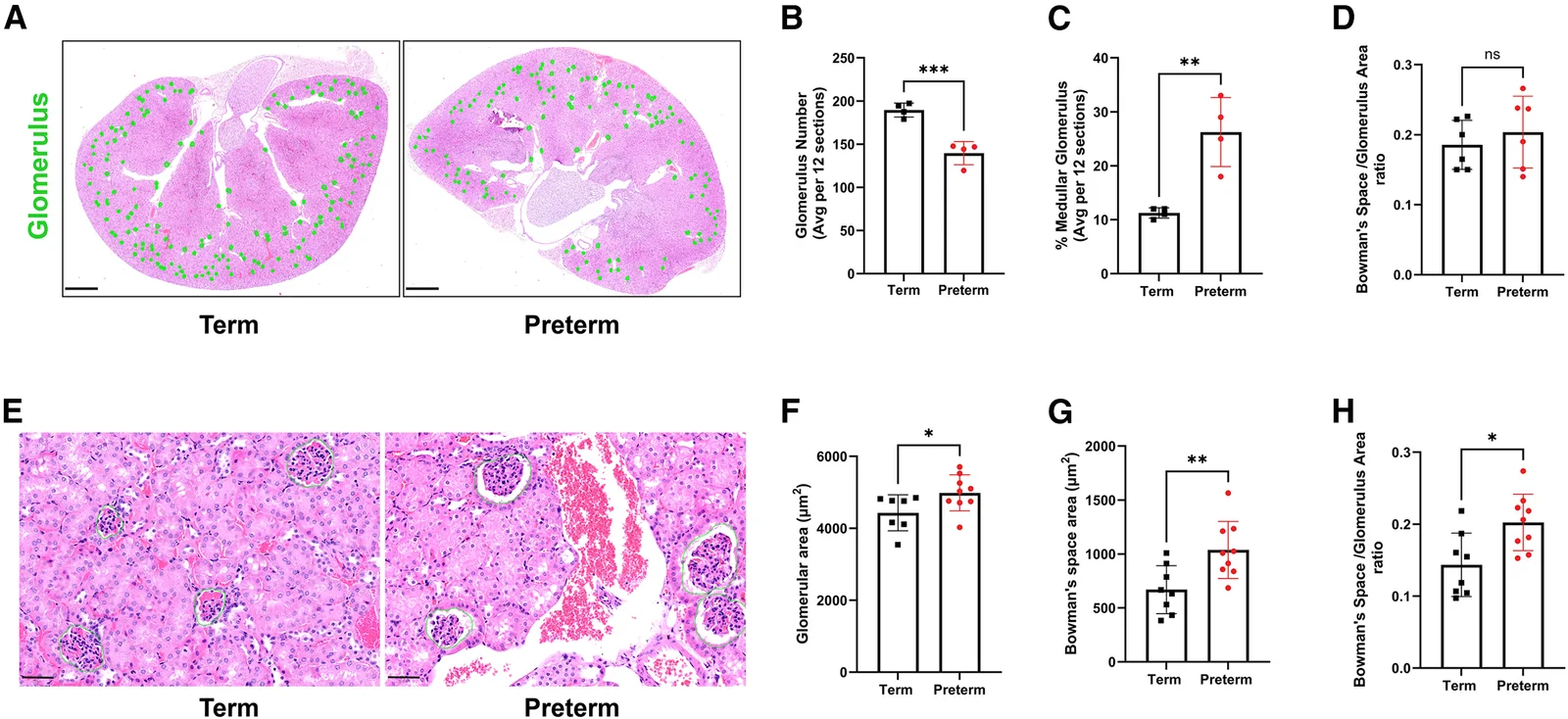
Preterm birth induces progressive glomerular structural alterations (A) Representative images of H&E kidney sections of term and preterm groups at P30. Scale bars, 200 μm. (B) Glomerulus number per section from term and preterm kidney sections at P30. *n* = 3–5 biological replicates per group (kidneys from independent litters). Each dot represents the mean of 12 technical replicates (non-adjacent sections per kidney). (C) Percent of medullary glomeruli as in (B). *n* = 3–5 biological replicates per group; technical replicates averaged per kidney. (D) Bowman’s space to glomerulus area ratio as in (B). *n* = 6 biological replicates per group; technical replicates averaged per kidney. (E) Representative images of H&E kidney sections from term and preterm offspring at P180. Scale bars, 50 μm; magnification, ×20. (F) Glomerular area (μm^2^) at P180 as in (E). *n* = 5–9 biological replicates per group; each dot represents the mean of 12 technical replicates (sections per kidney). (G) Bowman’s space area (μm^2^) as in (F). *n* = 5–9 biological replicates per group; technical replicates averaged per kidney. (H) Bowman’s space to glomerulus area ratio as in (F). *n* = 5–9 biological replicates per group; technical replicates averaged per kidney. Data are presented as mean ± SEM. A two-tailed Student’s *t* test was used. ∗*p* < 0.05, *∗∗p* < 0.01, *∗∗∗p* < 0.001 versus term. P, postnatal day.

Finally, we examined whether the functional deficits were associated with morphological adaptations. At P30, preterm kidneys contained significantly fewer glomeruli per section (Figures 5A and 5B), with an altered spatial distribution favoring the medullary region over the cortex (Figure 5C), suggestive of aberrant nephron positioning.^49^ This finding is also consistent with a selective disruption of postnatal nephrogenesis, as lineage tracing studies localize embryonic nephrons to the juxtamedullary region, and postnatal nephrons to the cortex.^50^ Despite these differences, no significant changes were observed in the ratio of Bowman’s space to glomerular area at this stage (Figure 5D).

By P180, progressive structural maladaptation became evident. Preterm kidneys displayed marked glomerular hypertrophy, characterized by an increase in total glomerular area and a significant expansion of Bowman’s space (Figures 5E–5H and S6). The increased Bowman’s space-to-glomerular area ratio reflects ongoing glomerular stress and hyperfiltration. These structural changes align with clinical observations in individuals born preterm and support the conclusion that preterm birth initiates a cascade of maladaptive remodeling that persists into adulthood.^51^^,^^52^

## Discussion

Preterm birth is increasingly recognized not merely as an acute neonatal event but as a lifelong determinant of renal health. Epidemiological studies consistently link prematurity with reduced nephron endowment and an elevated risk of CKD and hypertension in adulthood.^13^ However, the cellular and molecular mechanisms driving this developmental programming have remained elusive, often confounded in clinical settings by comorbidities such as sepsis, nephrotoxic medications, and postnatal steroid administration.^53^

In this study, using a non-surgical, established mouse model of preterm birth,^27^ we examined the specific impact of premature extrauterine life on kidney development. We demonstrate that preterm birth selectively affects nephron progenitor cells, temporarily impairing differentiation and inducing cell stress programs. Despite compensatory increases in differentiation and an extension to the period of nephrogenesis, this dysregulation leads to sustained reduction in nephron endowment, a structural-functional mismatch, and progressive maladaptive remodeling that persists into adulthood. In our model, although nephrogenesis is transiently prolonged following preterm birth, this extension fails to rescue nephron number due to a profound dysregulation in the timing and coordination of NPC proliferation and differentiation. Because over 50% of nephron formation in mice occurs during the early postnatal period,^36^ the transient developmental halt triggered by premature extrauterine exposure results in an irreversible functional deficit. Although surviving progenitor cells eventually initiate a compensatory increase in differentiation by PC20.5, the critical developmental window is time-limited, preventing full recovery of absolute nephron number before the niche is depleted.

Specifically, our findings both parallel and extend previous studies utilizing surgical cesarean models, which reported premature NPC differentiation and increased susceptibility to secondary postnatal insults, such as hyperglycemia-induced glomerular alterations in adulthood.^19^^,^^20^ While in this study, whole-kidney bulk RNA-seq approach captured a broad organ-level response to preterm delivery, our use of a non-surgical hormonal model combined with FACS-isolated NPC-specific transcriptomics allows us to pinpoint the unfolded protein response and ER stress specifically within the progenitor niche, minimizing the confounding inflammatory effects often associated with surgical trauma. Additionally, our findings complement those from neonatal hyperoxia models, which are often used to mimic the clinical oxygen support required by premature human infants.^21^ While exposure to hyperoxia in rats leads to long-term cardio-renal remodeling and tubular injury, the mice in our study were raised entirely in room air. This distinct experimental design demonstrates that the premature transition to the ex utero environment itself, even without the compounding oxidative stress of hyperoxia or surgical inflammation, represents a profound baseline insult sufficient to disrupt progenitor differentiation.

The transcriptional stress signatures observed in preterm NPCs at PC19.5 are uniquely driven by prematurity, rather than the general physiological stress of birth. Term NPCs analyzed at their equivalent ex utero transition stage (postnatal days 0–1, corresponding to PC19.5-PC20.5) did not exhibit the profound ER stress and unfolded protein response seen in the preterm group.

Together, our findings identify disrupted NPC temporal dynamics as a central mechanism linking prematurity to lifelong kidney vulnerability. Consistent with epidemiological and histopathological studies in human,^49^^,^^54^^,^^55^ preterm-born mice exhibited a significant nephron deficit by postnatal day 30, accompanied by elevated serum creatinine, urea, and albuminuria. Notably, these functional abnormalities manifested early and persisted into adulthood, despite increased body weight, underscoring that altered renal development rather than systemic growth restriction underlies the observed phenotype. Similar renal dysfunction has been reported in human preterm cohorts during childhood and adolescence, including higher blood pressure, albuminuria, and reduced GFR.^55^ Our findings complement these clinical observations by providing direct experimental evidence for causal developmental mechanisms.

We also observed a sexual dimorphism in renal susceptibility, with preterm males exhibiting significant nephron loss and long-term functional decline, while females were relatively protected, consistent with previously reported studies demonstrating male vulnerability to adverse perinatal conditions across organ systems.^56^ This sexual dimorphism, potentially driven by hormone-dependent mechanisms, warrants further investigation, particularly given its implications for long-term CKD risk stratification in preterm-born individuals.

A key and unexpected finding of this study is that preterm birth extends postnatal NPC persistence for approximately 24 h beyond the typical endpoint of nephrogenesis. However, this persistence is functionally ineffective. While the lifespan of NPCs may be intrinsically programmed to persist for their designated developmental duration,^26^ the acute stress of a premature delivery disrupts their functional efficiency. Li et al.^26^ demonstrated that excess fetal GDNF prolongs postnatal nephrogenesis, but as in our preterm model, this extension fails to rescue overall nephron endowment due to earlier inefficient nephrogenesis and dysregulated progenitor proliferation.

Our data reveal a dysregulated developmental trajectory after preterm birth at PC19.5, characterized by a transient disruption in NPC differentiation. By PC20.5, a compensatory surge in NPC proliferation, recovery of Six2^high^/LHX1^high^ NPCs, and transcriptional equivalence with term NPCs indicate partial restoration of nephrogenic programs. Nevertheless, sustained ER stress and NPC persistence indicate a lasting disruption that ultimately results in nephron deficit. These findings align with reports of continued, albeit often inefficient, postnatal nephrogenesis in human and primate preterm neonates,^49^^,^^51^^,^^57^ and with transient NPC disruptions leading to lasting nephron deficits.^19^^,^^58^

Mechanistically, our transcriptomic and biochemical analyses of FACS-isolated GFP^+^ NPCs revealed that preterm birth induces early transcriptional disturbances in NPCs, particularly at PC19.5. We identified a distinct stress signature in preterm NPCs as early as PC19.5, characterized by the upregulation of stress-related pathways, including ER stress, metabolic perturbations, and activation of the unfolded protein response, concomitant with suppression of differentiation-associated gene networks. These transcriptional alterations are corroborated by increased protein expression of ER stress markers, including BiP and PERK. Given that ER stress and UPR signaling have been shown to interface with molecular circuits regulating proliferation, differentiation, and apoptosis in embryonic stem cells and other progenitor populations,^59^^,^^60^ we propose that activation of this stress pathway in preterm NPCs may compromise the precise execution of developmental programs, thereby predisposing them to mistimed or inefficient differentiation.

The long-term consequences of these early developmental perturbations are substantial. At P180, preterm offspring developed albuminuria, elevated kidney injury biomarkers, and glomerular abnormalities, including Bowman’s space expansion and altered glomerular architecture. These structural changes resemble features of glomerular hyperfiltration and compensatory hypertrophy well-established sequelae of reduced nephron endowment,^61^ which mirrors human outcomes, where individuals born preterm often develop early hypertension, microalbuminuria, and increased CKD risk in adulthood.^62^ Thus, early-life disruption of NPC dynamics initiates a cascade of structural and functional alterations that extend well beyond the nephrogenic period.

The long-term functional impairment observed in our model closely parallels the clinical trajectory of human prematurity. For instance, the increased adult body weight in our preterm cohort reflects the well-documented phenomenon of early postnatal catch-up growth.^63^^,^^64^ Furthermore, while the outbred CD1 genetic background inherently introduces biological variance in parameters such as 24-h urinary albumin concentration, the animals exhibited no overt signs of systemic illness. Despite this expected variance, the significant and consistent elevation of multiple independent biomarkers—including serum NGAL, creatinine, and specific histological TIM1 upregulation provides robust, multi-modal evidence of chronic tubular injury resulting from preterm birth.

The strength of our study lies in the specific model utilized. Unlike surgical models of uterine ischemia or distinct genetic manipulations, the mifepristone-induced delivery mimics the spontaneous onset of preterm labor without direct fetal toxicity or inflammatory confounding. The fact that the term controls exposed to mifepristone showed no renal phenotype confirms that the defects are intrinsic to the premature exposure to the extrauterine environment. This isolation of prematurity suggests that even in the absence of severe neonatal complications, like AKI or sepsis, the premature kidney is biologically vulnerable.

In conclusion, we define a sequence of events linking preterm birth to adult kidney disease: environmental stress triggers a response in nephron progenitors that uncouples their proliferation from differentiation. This leads to a permanent nephron deficit that drives compensatory hyperfiltration and progressive glomerular injury. These findings highlight the perinatal period as a critical therapeutic window and suggest that interventions aimed at restoring proper nephron endowment could improve long-term renal outcomes in preterm infants.

### Limitations of the study

Our study has several limitations. First, while the mouse is a valuable model for mammalian kidney development, there are inherent species differences: notably, murine nephrogenesis continues postnatally,^30^ whereas human nephrogenesis is completed *in utero* in term infants.^11^^,^^12^ However, our model specifically mimics the human preterm condition, in which nephrogenesis is active at birth, and the fetus is exposed to the extrauterine environment. Second, our model does not account for the complex clinical care in the NICU (e.g., ventilation, antibiotics, and fluids), which may exacerbate renal injury in human neonates. Nevertheless, this limitation is also a strength, as it allows us to dissect the effects of prematurity per se, unconfounded by iatrogenic factors. Third, although our data demonstrate a strong temporal association between activation of the unfolded protein response and altered NPC dynamics, we did not perform functional rescue experiments. Future studies utilizing pharmacological modulators of ER stress, such as tauroursodeoxycholic acid (TUDCA) or 4-phenylbutyric acid (4-PBA)^65^^,^^66^ are warranted to definitively establish causality. It is important to acknowledge that our western blot analyses were performed on whole-kidney lysates; however, the absence of stress signatures in our non-NPC RNA-seq fractions strongly supports a localized progenitor response. Finally, due to the high biological variance in stereological counting at these specific early time points, we could not precisely quantify the exact number of nascent nephrons formed exclusively during this extended 24-h window. However, the ultimate permanent nephron deficit observed at P30 strongly suggests that this extended retention is largely inefficient.

## Resource availability

### Lead contact

Further information and requests for resources and reagents should be directed to and will be fulfilled by the lead contact, Morris Nechama (moris@hadassah.org.il).

### Materials availability

This study did not generate new unique reagents.

### Data and code availability

• Bulk RNA-seq data are publicly available as of the date of publication. Accession numbers are listed on the key resources table.
• All original analysis code has been deposited in GitHub and is publicly available as of the date of publication. Accession numbers are listed on the key resources table.
• Any additional information required to reanalyze the data reported in this paper is available from the lead contact upon request.

## Acknowledgments

This work was supported by grants from the 10.13039/501100003977Israel Science Foundation, Israel (2030/21 [to M.N.], 1394/25 and 2825/22 [to O.V.]), the Research Authority of Hadassah Hebrew University Medical Center, Israel (to O.V.). This work was also supported by 10.13039/501100020909AUSiMED (research grant to O.V., M.N. and A.N.C.), with additional support from the 10.13039/501100000925National Health and Medical Research Council of Australia (APP1156567 to A.N.C.), and the 10.13039/501100000923Australian Research Council (DP230103097 to A.N.C.). We thank Dr. Abed Nasereddin, Dr. Idit Shiff and Dr. Yariv Maron from the Core Research Facility, Faculty of Medicine, Hebrew University of Jerusalem, for assistance with RNA sequencing sample preparation and imaging analysis. We also acknowledge the use of facilities and technical support provided by Monash Micro Imaging and the Monash Bioinformatics and Genomics Platform.

## Author contributions

A.A., M.N., A.N.C., and O.V. conceived the study, designed the experiments, and wrote the manuscript. A.A. designed and conducted the experiments. M.P. performed the bulk RNA-seq bioinformatic analysis, generated all related figures, and submitted the data to GEO. J.L.M.M. assisted with imaging data analysis. A.A., A.S., A.N.C., O.V., and M.N. drafted the manuscript. All authors reviewed and approved the final manuscript.

## Declaration of interests

The authors declare no competing interests.

## Declaration of generative AI and AI-assisted technologies in the writing process

During the preparation of this work, the authors used ChatGPT and Gemini to rephrase the text. After using these tools, the authors reviewed and edited the content as needed and took full responsibility for the content of the publication.

## STAR★Methods

### Key resources table

**Table:** 

REAGENT or RESOURCE	SOURCE	IDENTIFIER
**Antibodies**		

Rabbit anti-Six2 antibody	Proteintech	Cat#11562-1-AP; RRID: AB_2189084
Mouse anti-Ki67 antibody (8D5)	Novus Biologicals	Cat#NBP2-22112; RRID: AB_3266719
Mouse anti-BrdU (Bu20a) antibody	Cell Signaling Technology	Cat#5292; RRID: AB_10548898
Mouse anti Cytokeratin antibody	Sigma-Aldrich	Cat#C2562; RRID: AB_476839
Mouse anti-TIM1 antibody	Abcam	Cat#AB78494; RRID: AB_1566803
Mouse anti-LIM1 (Lhx1) antibody (OTI2D5)	Thermo Fisher Scientific	Cat#MA5-25920; RRID: AB_2724601
Mouse anti-beta-actin antibody	Abcam	Cat#ab8226; RRID: AB_306371
Mouse Lotus Tetragonolobus Lectin antibody	Vector Laboratories	Cat# B-1325; RRID: AB_2336558
Cy3-conjugated goat anti-rabbit antibody	Jackson ImmunoResearch	Cat# 711-165-152; RRID: AB_2307443
Cy5-conjugated goat anti-mouse antibody	Bethyl Laboratories	Cat# A90-516C5; RRID: AB_10630988
Histofine Simple Stain MAX PO Anti-Mouse Anti-Rabbit Antibody	N-Histofine	Cat# 414152F

**Chemicals, peptides, and recombinant proteins**		

Mifepristone	Sigma-Aldrich	Cat#M8046
Bromodeoxyuridine (BrdU)	Sigma-Aldrich	Cat#B5002
DAB reagent	Thermo-Scientific	Cat#TA-125- QHDX
Hank’s Balanced Salt Solution	Sigma Aldrich	Cat#H6648-500ML
Collagenase	Sigma Aldrich	Cat#11213865001
DispaseII	Sigma Aldrich	Cat#D4693
Protease/Phosphatase inhibitors	MERCK	Cat#4906837001
DAPI	Sigma-Aldrich	Cat#D9542

**Critical commercial assays**		

QuantiChrom Urea assay kit	BioAssay Systems	Cat#DIUR-100
Mouse Creatinine kit	Crystal Chem	Cat#80350
QuantiChrom BCG Albumin Kit	BioAssay Systems	Cat#DIAG-250
Mouse NGAL ELISA kit	Crystal Chem	Cat#80655
*In situ* Cell Death Detection Kit, Fluorescein	Roche	Cat#11684795910
ER Stress Antibody Sampler Kit	Cell Signaling Technology	Cat#9956
Qiagen RNeasy Micro Kit	Qiagen	Cat#74004
qScript cDNA Synthesis Kit	Quantabio	Cat#95047
RNA ScreenTape kit	Agilent Technologies	Cat#5067-5576
Qubit® DNA HS Assay kit	Invitrogen	Cat#Q32852
SMARTer® Stranded Total RNA-Seq Kit v4	Takara Bio	N/A
NovaSeq 6000 SP Reagent kit v1.5 cartidge	Illumina	N/A
FOXP3 Fix/Perm Buffer Set	BioLegend	Cat#421403
LYNX Rapid RPE Antibody Conjugation Kit	Bio-Rad	Cat#LNK023RPE
LYNX Rapid APC Antibody Conjugation Kit	Bio-Rad	Cat#LNK032APC

**Deposited data**		

Bulk RNA-sequencing data from nephron progenitor cells from term and preterm at PC19.5 and PC20.5	This paper	GEO: GSE316576
Image analysis classifiers for immunofluorescence staining	This paper	GitHub: (GitHub - atharamleh-1/Prematurity)

**Experimental models: Organisms/strains**		

Mouse: CD1 outbred	Harlan Laboratories Israel	https://hlisrael.com/
Mouse: CD1*Tg (Six2-EGFP/cre) 1Amc*	Gift from Raphael Kopan’s lab, Developmental biology department, Cincinnati Children’s Hospital Medical Center, Ohio, USA	N/A

**Oligonucleotides**		

Primers for Mouse Six2 see Table 1	Sigma-Aldrich	See Table 1 for Batch# for Forward and Reverse
Primers for Mouse Lhx1 see Table 1	Sigma-Aldrich	See Table 1 for Batch# for Forward and Reverse
Primers for Mouse BMP2 see Table 1	Sigma-Aldrich	See Table 1 for Batch# for Forward and Reverse
Primers for Mouse Jag1 see Table 1	Sigma-Aldrich	See Table 1 for Batch# for Forward and Reverse
Primers for Mouse Wnt9b see Table 1	Sigma-Aldrich	See Table 1 for Batch# for Forward and Reverse
Primers for Mouse Wnt4 see Table 1	Sigma-Aldrich	See Table 1 for Batch# for Forward and Reverse

**Software and algorithms**		

LSRII flow cytometry and FlowJo software	The core research facility, The Hebrew University of Jerusalem, Jerusalem, Israel	N/A
IMARIS 9.8.0 software	The core research facility, The Hebrew University of Jerusalem, Jerusalem, Israel	N/A
Flow cytometry-based cell sorting	The core research facility, The Hebrew University of Jerusalem, Jerusalem, Israel	N/A
Qupath software	The core research facility, The Hebrew University of Jerusalem, Jerusalem, Israel	https://qupath.github.io/
GraphPad Prism Version 11.0.0 (93)	Pediatric Nephrology Research lab, Hadassah Medical School, Jerusalem, Israel.	https://www.graphpad.com/
Adobe illustrator 2026	Pediatric Nephrology Research lab, Hadassah Medical School, Jerusalem, Israel.	https://www.adobe.com/
Adobe Photoshop 2026	Pediatric Nephrology Research lab, Hadassah Medical School, Jerusalem, Israel.	https://www.adobe.com/
Fastqc Toolkit v0.11.5	Andrews S et al.^67^	https://www.bioinformatics.babraham.ac.uk/projects/fastqc
Trim Galore v0.6.10	Fóthi, Ábel et al.^68^	N/A
BBDuK	Nabakooza, Grace et al.^69^	N/A
STAR (2.7.10a)	Dobin, A. et al.^70^	N/A
FeatureCounts (v2.0.1)	Liao, Y. et al.^71^	N/A
MultiQC	Ewels, P. et al.^72^	N/A
edgeR	Robinson, Mark D. et al.^73^	N/A
Gprofiler	Kolberg, L. et al.^74^	N/A
DAVID	Sherman, B.T. et al.^75^	N/A
GSEA	Subramanian, A. et al.^76^	N/A
Custom image analysis scripts for immunofluorescence quantification	This paper	GitHub: (GitHub - atharamleh-1/Prematurity)

**Other**		

Mouse transcriptome and genome (GRCm39) with annotations	GRCm39	Ensembl release 109 annotations
E18.5 mouse kidney scRNA-seq dataset	Combes, A. N. et al.^41^	N/A

### Experimental model and study participant details

#### Animals

All mice were maintained at the Hebrew University Specific-Pathogen-Free (SPF) Animal Facility Units. All experiments were conducted using CD1 outbred mice purchased from Harlan Laboratories. Transgenic mice on the same CD1 outbred background were generously provided as a gift from the laboratory of Raphael Kopan, Department of Developmental Biology, Cincinnati Children's Hospital Medical Center, Ohio, USA. For all mice, the pregnancy date was determined by vaginal plug expulsion. The morning of plug detection was designated as the postconceptional day (PC) 0.5 of pregnancy. Pregnant females were randomly assigned to one of two groups: (i) preterm birth group, in which pregnant females received a single subcutaneous (SC) injection of 150 μg/kg of mifepristone (50 mg/ml in ethanol) (M8046; Sigma-Aldrich) at the PC17.5 to induce labor at PC18.5; and (ii) term control group, pregnant females were injected with the same dose at PC18.5 to induce normal delivery at PC19.5. The pups were dissected, and the kidneys were excised at PC days 19.5, 20.5, 21.5, and 22.5, or maintained until postnatal days (P) 30 and 180. At the designated age, body weight and combined kidney weight were recorded, and in some experiments, blood was drawn, 24-hour urine was collected, and the kidneys were excised.

When stated, we used the transgenic mice line *Tg(Six2-EGFP/cre)1Amc* (herein *Six2 Cre*^*+/tg*^). Heterozygous *Six2 Cre*^*+/tg*^ embryos were generated by mating 6–8-week-old *Six2 Cre*^*+/tg*^ male mice with WT CD1 females and used to sort GFP^+^ NPCs and GFP^-^ NPCs. In some experiments, Bromodeoxyuridine (BrdU) (B5002; Sigma-Aldrich) was diluted in PBS and injected SC (30ug/g) into preterm and term pups at PC19.5, PC20.5 and PC21.5 for 2 hours before being sacrificed, and the kidneys were excised for immunostaining.

#### Study approval

All animal experiments were approved by the Institutional Animal Care and Use Committee (IACUC) of the Hebrew University of Jerusalem (Protocol Number: MD-22-17110-3), and the study was conducted and reported in accordance with the ARRIVE guidelines.

### Method details

#### Biochemical analysis

Serum from the indicated mice in each group was collected and analyzed for serum urea using the QuantiChrom Urea assay kit (DIUR-100; BioAssay Systems), serum creatinine using Crystal Chem Mouse Creatinine kit (80350; Crystal Chem), and for serum NGAL level using Crystal Chem Mouse NGAL ELISA kit (80655; Crystal Chem). For 24-hour urine collection, mice from each group were placed in special metabolic cages at the SPF animal facility at the Hebrew University of Jerusalem for 24 hours, and the urine was then centrifuged at 2500 RPM, 4 C for 10 minutes to discard any debris. The albumin urine level was performed using QuantiChrom BCG Albumin Kit (DIAG-250; BioAssay Systems).

#### Nephron count

Nephron count was performed as previously described.^28^^,^^29^ Briefly, at P30, a single kidney of the indicated mice was dissected. The kidneys were chopped into small pieces and digested in 5 mL of 6N HCl at 37°C for 90 minutes. The tissue was further dissociated by repeated pipetting. 25ml of ddH_2_O was added to each sample and kept at 4°C overnight. 200 μl of well-mixed suspension was placed in a 1 cm × 1 cm area of a P100 culture dish with gridlines to count the number of nephrons. The number of glomeruli was counted three times under an inverted microscope. The total number of glomeruli was estimated as follows: total nephron number per kidney equaled the average measured number of glomeruli in 30ml of the suspension.

#### Section preparation and immunostaining

Kidneys at indicated time points were dissected in ice-cold PBS and fixed in fresh 4% formaldehyde in PBS. Kidneys were embedded in paraffin and sectioned. H&E staining was performed as indicated to assess glomerular location and morphology (see below). Immunofluorescence (IF) /Immunohistochemistry (IHC) staining was performed as previously described.^77^ Briefly, paraffin-embedded tissue sections (4–6 μm) were deparaffinized, hydrated, and incubated overnight at 4°C with the following antibodies, according to the manufacturer's instructions: DAPI (D9542; Sigma-Aldrich), rabbit anti-Six2 antibody (11562-1-AP; Proteintech), mouse anti-Ki67 antibody (8D5) (NBP2-22112; Novus Biologicals), mouse anti-BrdU (Bu20a) antibody (5292; Cell Signaling Technology), mouse anti Cytokeratin antibody (C2562; Sigma-Aldrich), and mouse anti-TIM1 antibody (AB78494; Abcam), mouse anti-Lotus tetragonolobus lectin (LTL), (B-1325; Vector Laboratories). After extensive washing in PBS/0.5%Tween, for IF staining, the sections were incubated with Cy3-conjugated goat anti-rabbit (711-165-152; Jackson ImmunoResearch) or Cy5-conjugated goat anti-mouse (A90-516C5; Bethyl Laboratories) antibody according to the manufacturer’s instructions. For IHC, DAB reagent (TA-125-QHDX; Thermo-Scientific) was applied after incubation with Histofine Simple Stain MAX PO Anti-Mouse Anti-Rabbit Antibody (414152F; N-Histofine). Sections and slides were visualized with either a Nikon confocal A1R or a Nikon spinning disk confocal microscope. For apoptosis assessment, *In Situ* Cell Death Detection Kit, Fluorescein (11684795910; Roche) was used according to the manufacturer's instructions. For immunofluorescence and immunohistochemistry analyses, kidneys from three independent litters were analyzed per experimental group and postconceptional day (*n* = 3 biological replicates). For each kidney, three non-adjacent sections were stained and imaged under identical acquisition settings and were considered technical replicates. Quantification was performed on each section, and values from technical replicates were averaged to generate a single value per biological replicate prior to statistical analysis.

#### Glomerular morphology

Kidneys from term and preterm offspring at P30 and P180 were collected from independent litters. For each experimental group, 3–9 kidneys were analyzed (biological replicates). Each kidney was sectioned, and 12 non-adjacent sections per kidney were stained with H&E. Glomerular number, location, area, and Bowman’s space were measured in each section using Qupath software, and the mean of 12 non-adjacent sections (technical replicates) were used to represent a single biological replicate.

#### Fluorescence-activated cell sorting (FACS) of *Six2 Cre*^*+/tg*^ nephron progenitor cells

FACS-based NPCs isolation was performed as previously described.^78^ Briefly, Kidneys from term and preterm mice were collected at the postconceptional days 19.5 and 20.5. Kidneys were excised and immediately placed in an ice-cold Hank’s Balanced Salt Solution (HBSS) (H6648-500ML; Sigma Aldrich) buffer. The kidneys were sliced and chopped into ∼0.5-1 mm pieces on ice using a surgical scalpel. The chopped kidneys were transferred into an HBSS solution containing 1 μg/μl collagenase (11213865001; Sigma Aldrich) and dispase II (D4693; Sigma Aldrich) and incubated for 30 minutes at 37°C. The cells were filtered through a 40μm nylon cell strainer (352340; Corning) and washed twice with cold HBSS. For each time point, kidneys from 3–4 independent litters (9–15 pups per pooled sample) were pooled to isolate *Six2 Cre*^*+/tg*^ GFP^+^ NPCs and GFP^-^ non-NPC kidney cell by cell flow cytometry-based cell sorting (Hebrew University Faculty of Medicine Core Facility). For RNA extraction, 50,000-100,000 cells were washed in cold HBSS, and total RNA was extracted using the Qiagen RNeasy Micro Kit (74004; Qiagen).

#### RNA sequencing and qRT-PCR validation

Bulk RNA-sequencing and qRT-PCR validation were performed on FACS-isolated nephron progenitor cell samples as described above. Each sample represents an independent litter and comprises pooled NPCs from 9–15 animals of both sexes. RNA integrity was assessed using the Tape Station system using an RNA ScreenTape kit (5067-5576; Agilent Technologies). RNA was quantified using a Qubit apparatus (Qubit® DNA HS Assay kit Q32852; Invitrogen). Libraries were prepared from RNA samples using a SMARTer® Stranded Total RNA-Seq Kit v4 (Takara Bio). The libraries were barcoded and pooled for multiplex sequencing. The pooled DNA library was loaded on a NovaSeq 6000 SP Reagent Kit v1.5 (100 cycles) cartridge (Illumina) and sequenced on an Illumina Novaseq 6000 System using a single-read sequencing condition of 100 cycles. Library preparation and sequencing were performed at the Core Facility of the Hebrew University Faculty of Medicine. For further validation, RNA was extracted, reverse transcribed using the qScript cDNA Synthesis Kit (95047; Quantabio) and used for qRT-PCR with the primers listed in Table 1.

**Table 1:** Oligonucleotide sequences, related to STAR Methods

Batch#	Sequence (5'→3′)	Oligo name
RE00826406	CCGAGGCCAAGGAAAGGGAG	Mouse Six2 Forward
RE00826407	CACTGCCATTGAGCGAGGAAG	Mouse Six2 Reverse
RE00826408	CGACTCTAATTGCCCTGTCGC	Mouse Lhx1 Forward
RE00826409	TGGTACCGAAACATCGGAAGA	Mouse Lhx1 Reverse
RE00826410	TCCATCACGAAGAAGCCGTG	Mouse BMP2 Forward
RE00826411	TCGTCACTGGGGACAGAACT	Mouse BMP2 Reverse
RE00826412	AACTGTTGTGGTGGAGTCCG	Mouse Jag1 Forward
RE00826413	GCAAAGTGTAGGACCTCGGC	Mouse Jag1 Reverse
RE00832016	CTAGTGGCGCGAGGAGATG	Mouse Wnt9b Forward
RE00832017	GACCGGTCAGGCCGAAG	Mouse Wnt9b Reverse
RE00767826	TCTCACAGTCCTTTGTGG	Mouse Wnt4 Forward
RE00767827	CATGTGTGTCAAGATGGC	Mouse Wnt4 Reverse

#### Read processing, alignment, and quality control

Raw reads were assessed for quality using FastQC (v0.11.5),^67^ examining per-base sequence content, GC distribution, adaptor contamination, duplication levels, and overrepresented sequences. Based on these metrics, reads were processed using Trim Galore (v0.6.10),^68^ and BBDuK (v38.18),^69^ to remove adaptor sequences, overrepresented reads, and low-quality bases at both 5′ and 3′ ends.

Processed reads were aligned to the mouse reference genome (GRCm39) with Ensembl release 109 annotations using STAR (2.7.10a).^70^ Gene-level counts were quantified using featureCounts (v2.0.1)^71^ and alignment metrics were summarized with MultiQC.^72^

Sample relationships were assessed using multidimensional scaling (MDS) plots, alongside inspection of canonical nephron progenitor and differentiation markers. Outlier samples (S15, S17, S31) were identified based on discordant clustering and marker expression and excluded from downstream analyses.

#### Differential expression analysis

Differential expression analysis was conducted in R using the *edgeR*.^73^ Raw counts were converted to a DGEList object, lowly expressed genes were removed, and library-sizes were normalized using the trimmed mean of M-values (TMM) method. A group-based design matrix was used to model the experimental conditions, and dispersion parameters were estimated before fitting negative binomial generalized linear models.

Differential expression was assessed using the quasi-likelihood F-test (QLF), with genes considered significant at FDR < 0.05. Pairwise contrasts included comparisons between developmental stage and condition (e.g. term19 vs term20; preterm19 vs preterm20).

#### Functional enrichment and gene set analysis

Functional enrichment analysis of differentially expressed genes (FDR < 0.05) was performed using *DAVID* and *g:Profiler*.^74^^,^^75^ Gene set enrichment analysis (GSEA) was performed using the GSEA desktop application with gene sets from MSigDB, Panther, KEGG, and GO pathway collections.^76^ Directional pathway enrichment (e.g. Figure 3D) and comparisons of custom cell type signatures were performed using the g:Profiler multiquery framework (version e113_eg59_p19_6be52918; g:SCS correction).^74^

#### Custom cell type signatures

Custom gene signatures for NPCs and early proximal tubule, distal tubule and podocyte signatures were derived from a mouse scRNAseq reference.^41^ Marker genes were required to meet logFC > 0.5, FDR < 0.05, and cluster-specific expression. This approach excludes some canonical markers (e.g. *Six2*), which are expressed across nephron progenitor and early committing cell types (e.g. NPC, PTA, RV) but enables clearer discrimination between progenitor and early differentiated states. These custom signatures are provided in Data S2.

#### Western blotting

Kidneys, as indicated, were extracted and homogenized in cold RIPA buffer containing 150 mM NaCl, 1% NP-40, 0.5% sodium deoxycholate, 0.1% Sodium dodecyl sulfate (SDS), 25 mM Tris (pH 7.4), supplemented with protease/phosphatase inhibitors (4906837001; MERCK). An equal amount of protein extract was analyzed by SDS-PAGE as previously described,^78^ using the following antibodies: mouse anti-LIM1 (LHX1) antibody (OTI2D5) (MA5-25920; Thermo Fisher Scientific) and mouse anti-beta-actin antibody (ab8226; Abcam) according to the manufacturer's instructions. ER Stress Antibody Sampler Kit (9956; Cell Signalling Technology) was used according to the manufacturer's instructions.

#### Flow cytometry-based analysis

Kidneys were collected from term and preterm mice at the postconceptional days 19.5–21.5 and immediately placed in ice-cold Hank’s Balanced Salt Solution (HBSS) (H6648; Sigma Aldrich). Tissues were minced on ice into ∼0.5–1 mm fragment using sterile scalpels and then enzymatically dissociated in HBSS containing 1 μg/μl collagenase (11213865001; Sigma-Aldrich) and dispase II (D4693; Sigma-Aldrich) for 30 minutes at 37°C. The resulting suspension was passed through a 40 μm nylon strainer (352340; Corning) and washed twice with ice-cold HBSS. Single-cell suspensions (1 × 10^6^ cells) were fixed and permeabilized using the FOXP3 Fix/Perm Buffer Set (421403; BioLegend) according to the manufacturer’s protocol. Cells were stained with Six2 conjugated to R-phycoerythrin (PE) and LHX1 conjugated to allophycocyanin (APC), prepared using the LYNX Rapid RPE and APC Antibody Conjugation Kits (LNK023RPE, LNK032APC; Bio-Rad), respectively. Samples were analyzed on an LSR II flow cytometer (BD Biosciences), and data were processed using FlowJo software (BD Biosciences). For each experimental group and postconceptional day, kidneys from three independent litters were used (*n* = 3 biological replicates per postconceptional day per group).

### Quantification and statistical analysis

#### Image analysis

Images were analyzed using Imaris or QuPath software. For co-staining analyses conducted in either Imaris or QuPath, the same two-step workflow was applied to specifically assess staining in nephron progenitor cells (NPCs). First, NPCs were identified based on Six2 staining. Next, either the number of double-positive cells or the intensity of the second antibody signal was quantified. All measurements were normalized to the NPCs total number. For TIM1 image analysis, QuPath was used. Tissue regions were identified using H&E staining, and TIM1 expression was quantified based on DAB signal intensity. All analysis scripts and classifiers are openly available on GitHub: (GitHub - atharamleh-1/Prematurity). Quantification of proliferating proximal tubular epithelial cells was performed as follow: Proximal tubular regions were identified based on Lotus tetragonolobus lectin (LTL) staining. LTL-positive tubules were automatically annotated to generate regions of interest encompassing the entire tubular epithelial compartment, including the associated nuclei. Within the LTL-defined tubular annotations, nuclei were detected using DAPI staining. Ki67-positive proliferating nuclei were identified based on co-localization of Ki67 immunoreactivity with DAPI-positive nuclei. Only nuclear Ki67 signal within LTL-positive tubular annotations were included in the analysis. The number of Ki67^+^/DAPI^+^ nuclei within LTL-positive tubular annotations was normalized to the total number of DAPI-positive nuclei within the same LTL-positive regions and expressed as the percentage of proliferating proximal tubular cells. All quantitative analyses were performed on biological replicates, with technical replicates averaged prior to statistical testing.

#### Statistical analysis

The numbers of biological samples were determined based on effect size or sample variation. No statistical method was used to predetermine the sample size. RNA-seq samples were assessed for quality and consistency using standard QC metrics and exploratory analyses (including distributional assessment/PCA). Samples identified as outliers (out-of-range values relative to the cohort structure) were excluded prior to downstream analyses. Animals were randomly assigned to groups for *in vivo* studies; no formal randomization method was applied when assigning animals for treatment. Values are presented as means ± SEM. Statistical significance was determined using a two-tailed Student's t-test or one-way ANOVA as indicated, (∗*p* < 0.05, ∗∗*p* < 0.01, ∗∗∗*p* < 0.001) using GraphPad Prism analysis software.

## Supplemental information

Document S1. Figures S1–S6

Data S1. Differential expression results from Six2-GFP + NPCs presented in Amleh et al. NPCs were isolated from term and preterm groups at post-conceptional days 19.5 and 20.5, related to Figures 3 and S4

Data S2. E18 Mouse early nephron markers unique.gmt, the custom NPC and early nephron signatures used for gene set testing, related to STAR Methods

Data S3. Uncropped western blots, related to STAR Methods
