# Supplementary material for: Preterm birth disrupts nephron progenitor cell dynamics predisposing to renal disease
Source: iScience. 2026 Sep 8;29(9):117482. doi: 10.1016/j.isci.2026.117482 (PMC13573688; doi:10.1016/j.isci.2026.117482)
Supplement: Supplementary file 1 [file mmc1.pdf]

iScience, Volume *29*

Supplemental information

Preterm birth disrupts nephron progenitor cell dynamics predisposing to renal disease

Athar Amleh, Mehran Piran, Julie L.M. Moreau, Aviad Schnapp, Alexander N. Combes, Oded Volovelsky, and Morris Nechama

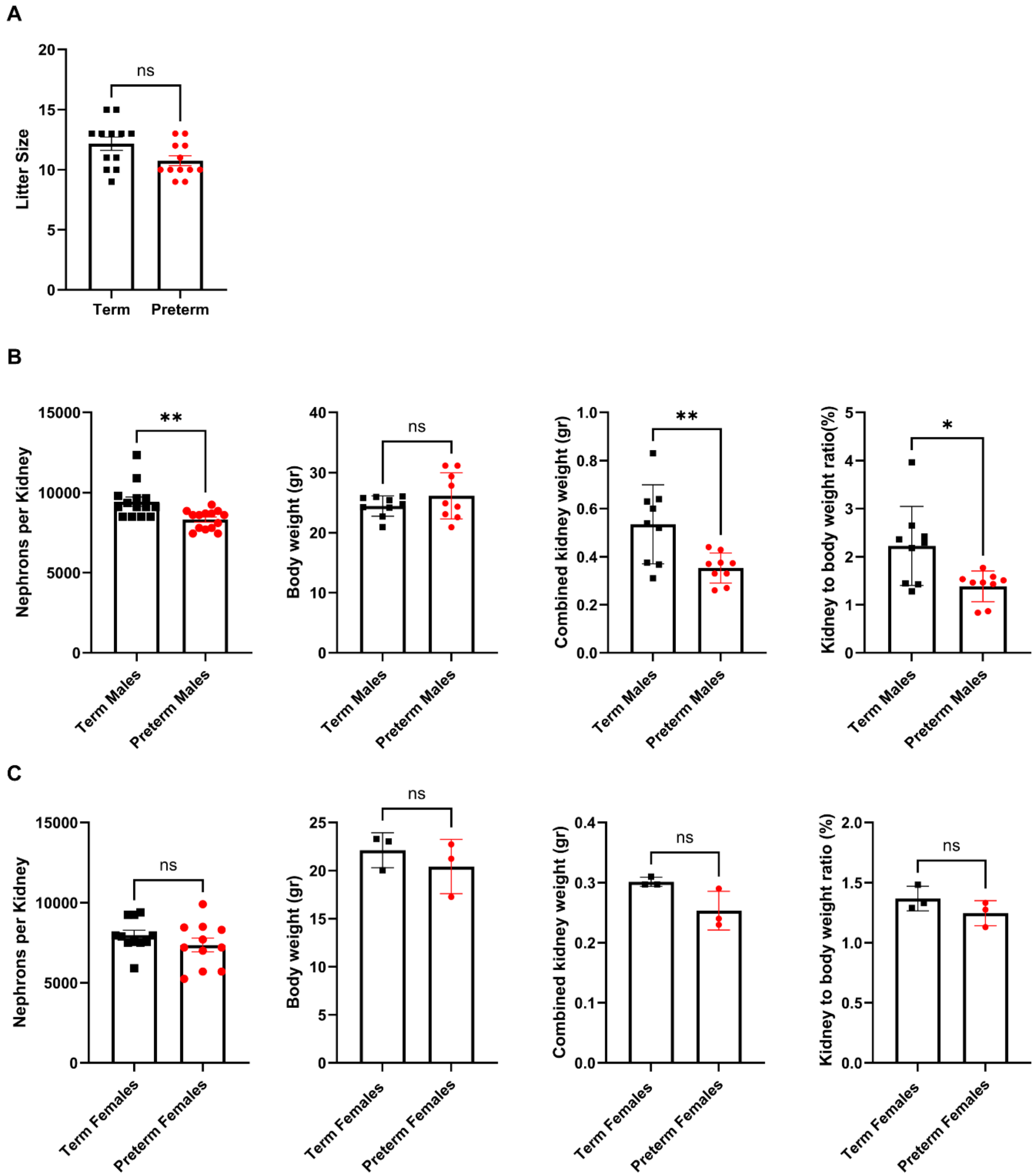

Figure S1 **Preterm birth did not affect litter size but revealed male-specific differences in kidney and body parameters, Related to Figure 1**

(A) litter size at P0 in pups from term and preterm groups. n= 10-12 pups from three independent litters per group (biological replicates).

(B) Male offspring: nephron number at P30 determined by acid maceration, body weight, combined kidney weight, and kidney-to-body weight ratio. n = 15–20 pups from three independent litters per group (biological replicates). **(C)** Female offspring: nephron number at P30 determined by acid maceration, body weight, combined kidney weight, and kidney-to-body weight ratio. n = 11–15 pups (nephron number) and 3–5 pups (body/kidney weight measurements) from three independent litters per group (biological replicates). Data are presented as mean ± SEM. A two-tailed Student's t-test was used**.** * $P < 0.05$, ** $P < 0.01$, *** $P < 0.001$ versus term. P, postnatal day.

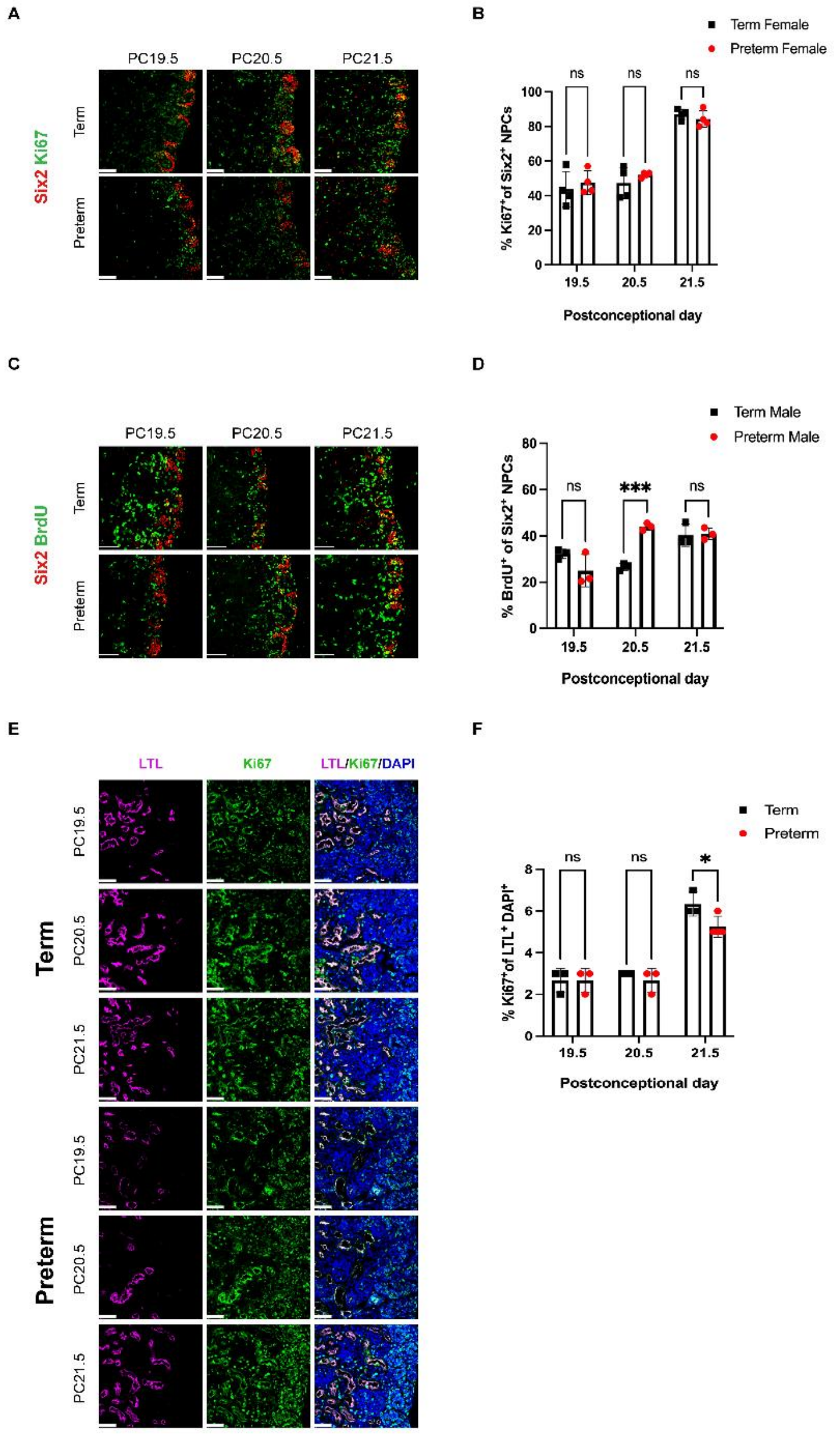

Figure S2 <u>**Preterm birth altered nephron progenitor cell proliferation in males without affecting other kidney cell types, Related to Figure 2**</u>

(A) Representative images of Kidney sections from female term and preterm offspring at PC19.5 to PC21.5, stained for Six2 (NPC marker) and Ki67 (proliferation marker). Scale bars, 50 µm; magnification, x20. **(B)** Quantification of $Ki67^+$ proliferating NPCs shown in (A) n = 3 biological replicates per group per PC (kidneys from independent litters). Each dot represents the mean of three technical replicates (non-adjacent sections per kidney). **(C)** Representative images of Kidney sections from male term and preterm offspring at PC19.5 to PC21.5, stained for Six2 (NPCs marker) and BrdU (proliferation marker). Scale bars, 30 µm; magnification, x20. Pups were injected with BrdU (30 µg/g) for 2 hours prior to kidney extraction. **(D)** Quantification of $BrdU^+$ proliferating NPCs shown in (C) n = 3 biological replicates per group per PC (kidneys from independent litters). Each dot represents the mean of three technical replicates (non-adjacent sections per kidney). (**E**) Representative images of Kidney sections from term and preterm offspring at PC19.5 to PC21.5, stained for LTL (Proximal tubule cell marker) and Ki67 (proliferation marker). Scale bars, 50 µm; magnification, x20. **(F)** Quantification of $Ki67^+$ proliferating proximal tubule cells shown in (E) n = 3 biological replicates per group per PC (kidneys from independent litters). Each dot represents the mean of three technical replicates (non-adjacent sections per kidney). Data are presented as mean ± SEM. One-way ANOVAs were used. * $P < 0.05$, ** $P < 0.01$, *** $P < 0.001$. PC, postconceptional day

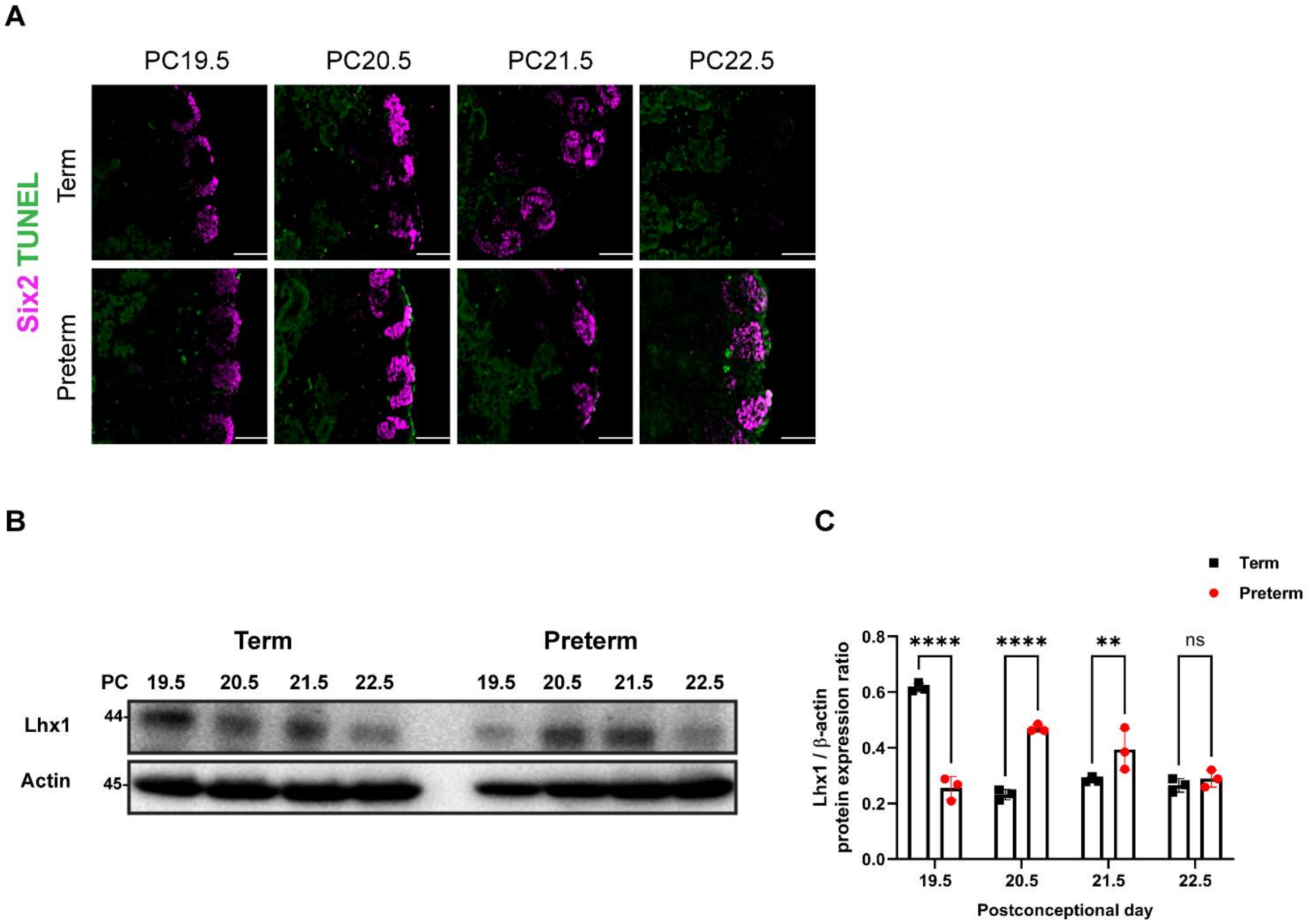

Figure S3 __Preterm birth altered nephron progenitor cell differentiation without affecting apoptosis, Related to Figure 2__

(**A**) Representative images of Kidney sections from term and preterm offspring at PC19.5 to PC22.5, stained for Six2 (NPC marker) and TUNEL (apoptotic cell marker). Scale bars, 30 µm; magnification, x40. **(B)** Western blot analysis for LHX1 and actin protein expression in term and preterm kidney extracts at PC19.5-PC22.5. **(C)** Quantification of the Western blots shown in (B). n = 3 biological replicates per group per PC (protein extracts prepared from kidneys of three independent litters). Each value represents a single biological replicate; no technical replicates were performed. Data are presented as mean ± SEM. One-way ANOVAs were used. * $P < 0.05$, ** $P < 0.01$, *** $P < 0.001$. PC, postconceptional day.

A

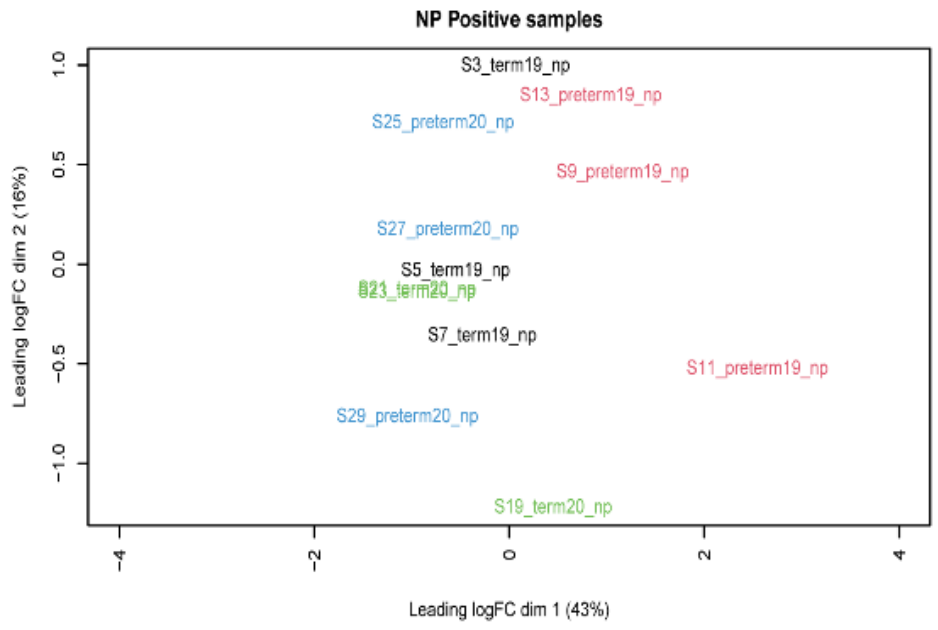

B

cluster_markers_unique_only		Up in T19 FDR<0.0	Up in PT19 FDR<0.	Up PT PC19.5 FDR	Up PT PC20.5 FDR
Term Name	Term ID	p_adj	p_adj	p_adj	p_adj
Unique marker genes for EPT (logFC>0.5, FDR<0.05, cl...	EPT	7.330×10^{-85}	1.000	1.000	3.560×10^{-78}
Unique marker genes for NP (logFC>0.5, FDR<0.05, clu...	NP	1.000	1.779×10^{-3}	8.367×10^{-5}	1.000

C

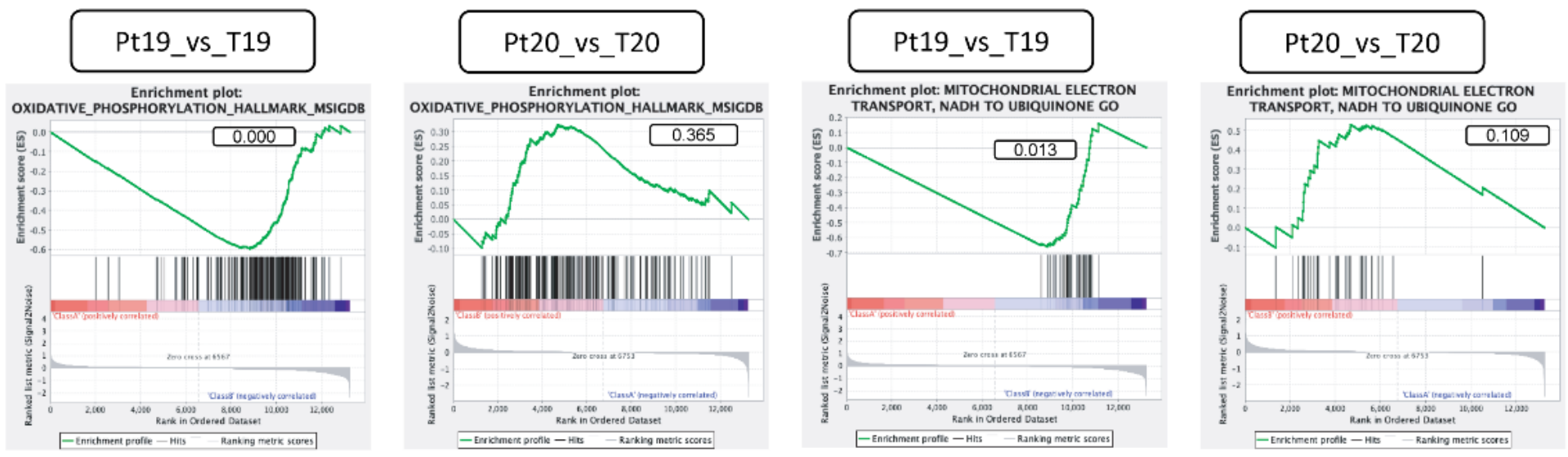

D

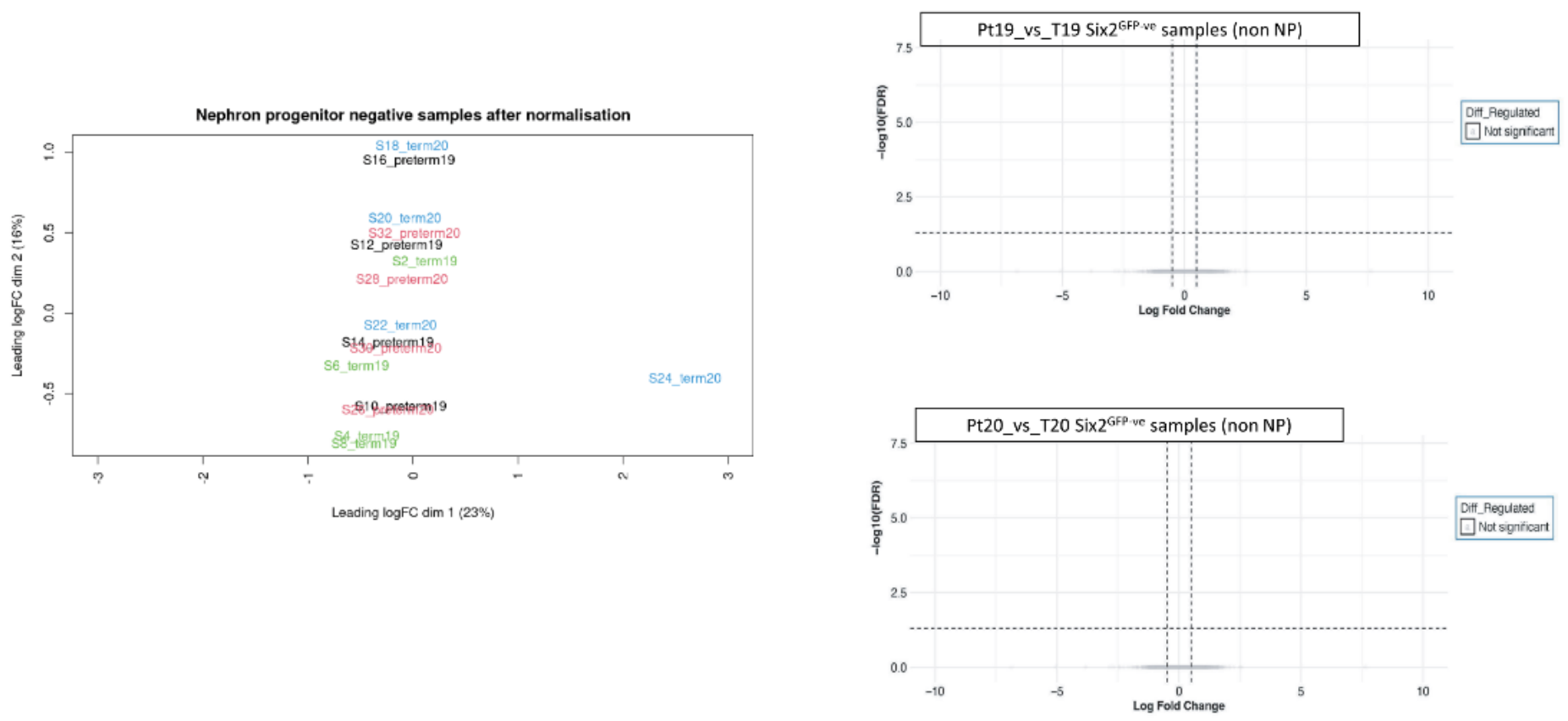

Figure S4 <u>Bulk RNA-seq analysis of Six2⁻ (nephron progenitor–negative; stromal-enriched) fetal kidney cell fractions from term and preterm mice, Related to Figure 3</u> (A) MDS plot of normalized transcriptomes from quality-controlled Six2GFP⁺ nephron progenitor (NP) samples across Term19, Preterm19, Term20, and Preterm20 groups. **(B**) Gene set testing showed enrichment of NPC self-renewal signatures in preterm PC19.5 NPCs (adjusted p value 1.779×10^{-3}), and early proximal tubule signatures were enriched in term PC19.5 (Adjusted p value 7.330×10^{-85}). **(C)** Gene set enrichment analysis (GSEA) comparing preterm versus term samples at stages 19 and 20 (Pt19_vs_T19 and Pt20_vs_T20), highlighting enrichment of oxidative phosphorylation (Hallmark) and mitochondrial electron transport from NADH to ubiquinone (GO) signatures; nominal P values are indicated on each plot. **(D)** MDS plot of normalized Six2GFP⁻ (non-NP) samples and volcano plots for differential expression in the Six2GFP⁻ fraction for Pt19_vs_T19 and Pt20_vs_T20 comparisons, illustrating limited transcriptional differences in the NP-negative/stromal compartment. For each time point, Six2GFP⁻ (non-NP) cells were isolated from the same pooled kidney samples used for Six2GFP⁺ RNA-seq (3–4 litters per pool, 9–15 pups total per pool). A total of n = 3-4 pooled biological replicates per group per time point were sequenced. No technical replicate libraries were performed.

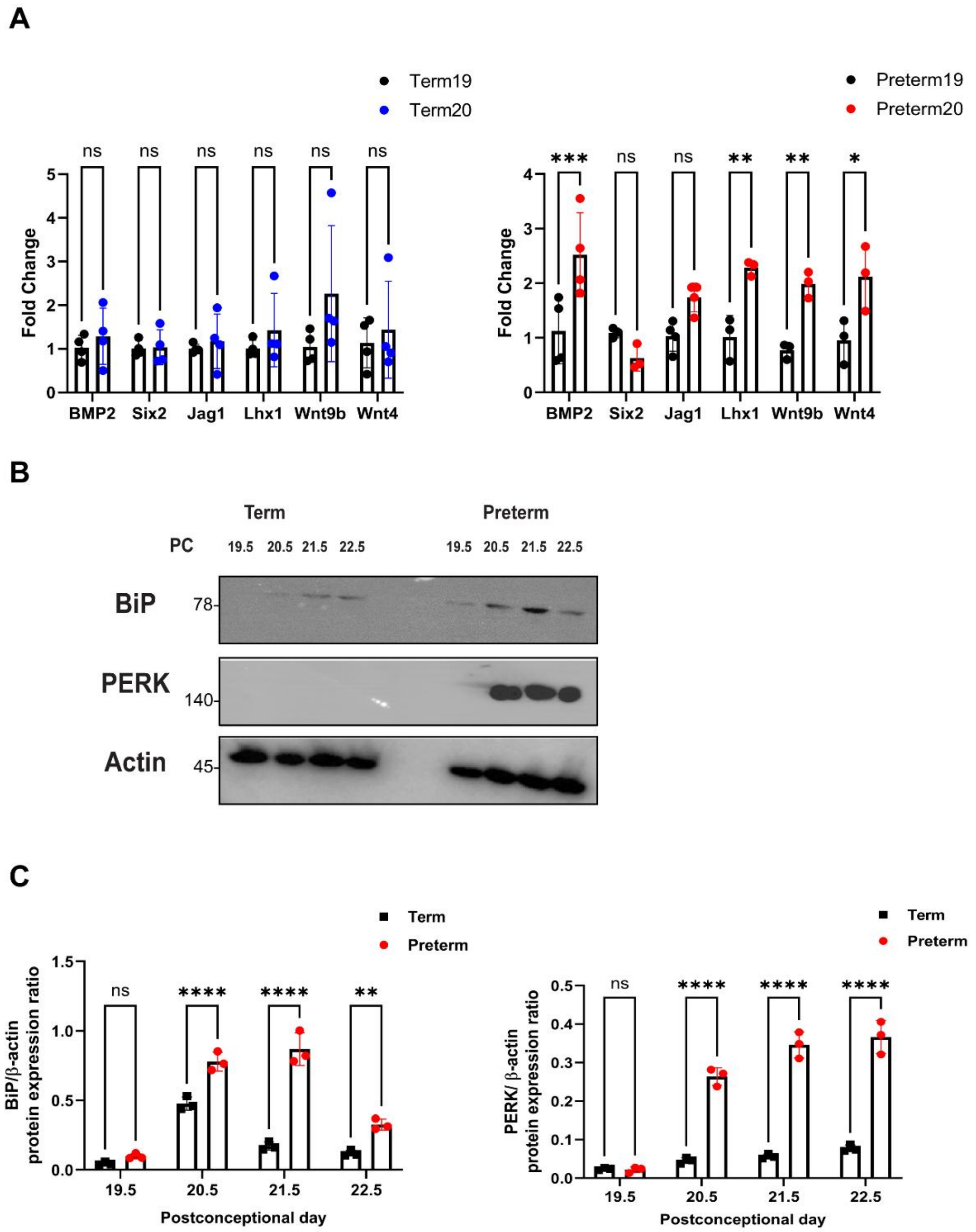

Figure S5 **Preterm birth is associated with altered regulation of nephron progenitor cell gene expression and increased ER stress, Related to Figure 3** **(A)** RT-qPCR analysis of genes associated with nephron progenitor cell self-renewal and differentiation at PC19.5 and PC20.5 in term and preterm groups. n = 3 biological replicates per group per time point (RNA isolated from the same kidney extracts used for RNA-seq). **(B)** Western blot analysis of the ER stress proteins BiP and PERK and β-actin protein expression in term and preterm kidney extracts at PC19.5 up to PC22.5. **(C)** Quantification of Western blots shown in (B). n = 3 biological replicates per group per time point (protein extracts prepared from kidneys of independent litters). Each value represents a single biological replicate; no technical replicate gels were performed. Data are presented as mean ± SEM. One-way ANOVAs were used. * $P < 0.05$, ** $P < 0.01$, *** $P < 0.001$. PC, postconceptional day.

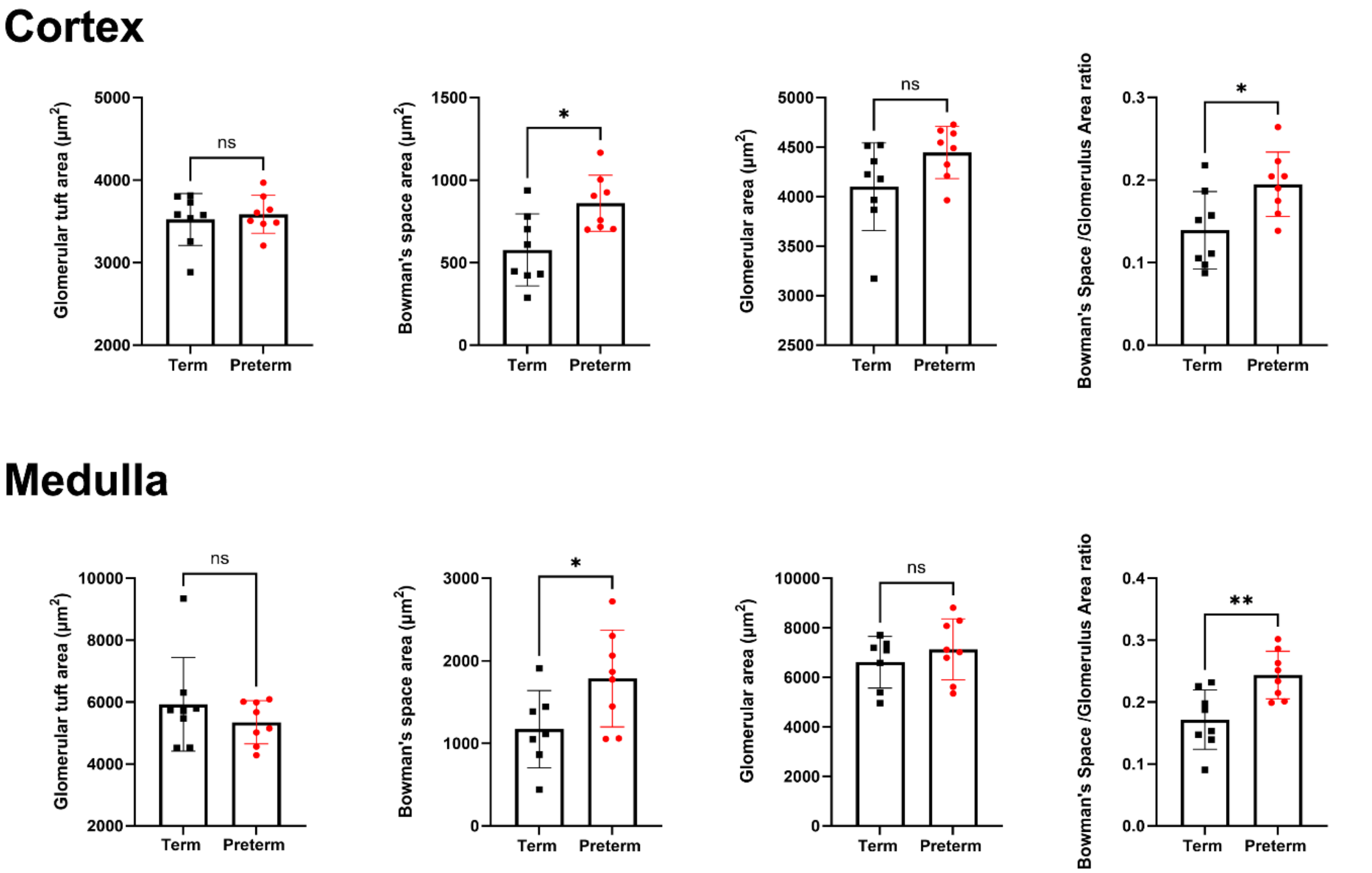

Figure S6 **<u>Progressive glomerular structural changes in cortical and medullary regions following preterm birth, Related to Figure 5</u>** Analysis of glomerular tuft area, Bowman's space area, glomerular area, and the Bowman's space-to-glomerular area ratio in term and preterm kidney sections at P180, analyzed separately in the cortex (upper panel) and medulla (lower panel). n = 6 biological replicates per group (kidneys from independent litters). For each kidney, 12 non-adjacent sections were analyzed, and the mean of the 12 sections was used to represent a single biological replicate. Data are presented as mean ± SEM. A two-tailed Student's t-test was used**.** * $P < 0.05$, ** $P < 0.01$, *** $P < 0.001$ versus term. P, postnatal day.
