# Supplementary material for: Preterm birth disrupts nephron progenitor cell dynamics predisposing to renal disease
Source: iScience. 2026 Sep 8;29(9):117482. doi: 10.1016/j.isci.2026.117482 (PMC13573688; doi:10.1016/j.isci.2026.117482)
Supplement: Supplementary file 2 [file mmc4.pdf]

Related to Figure S3

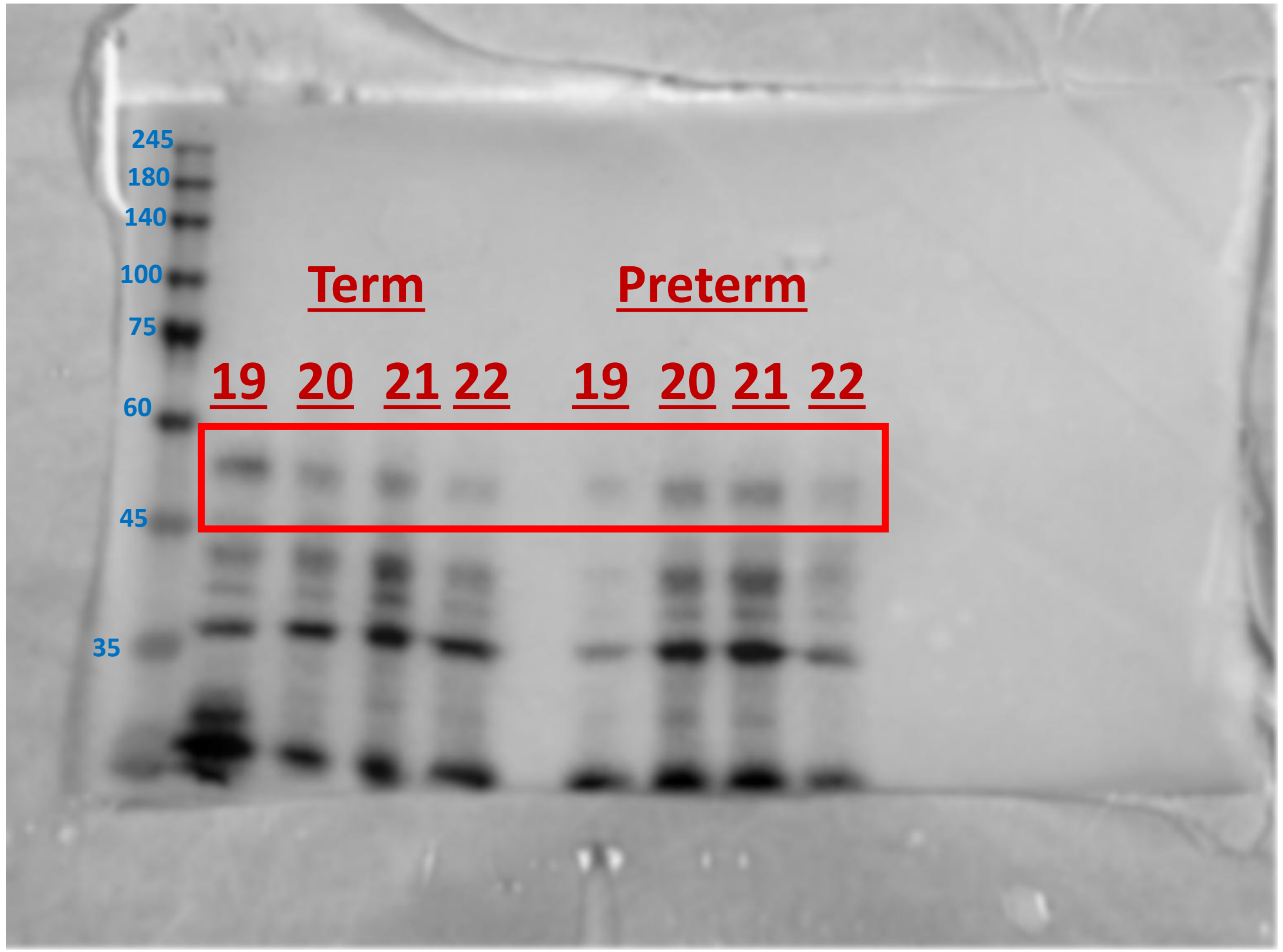

LHX1 44-55KDa

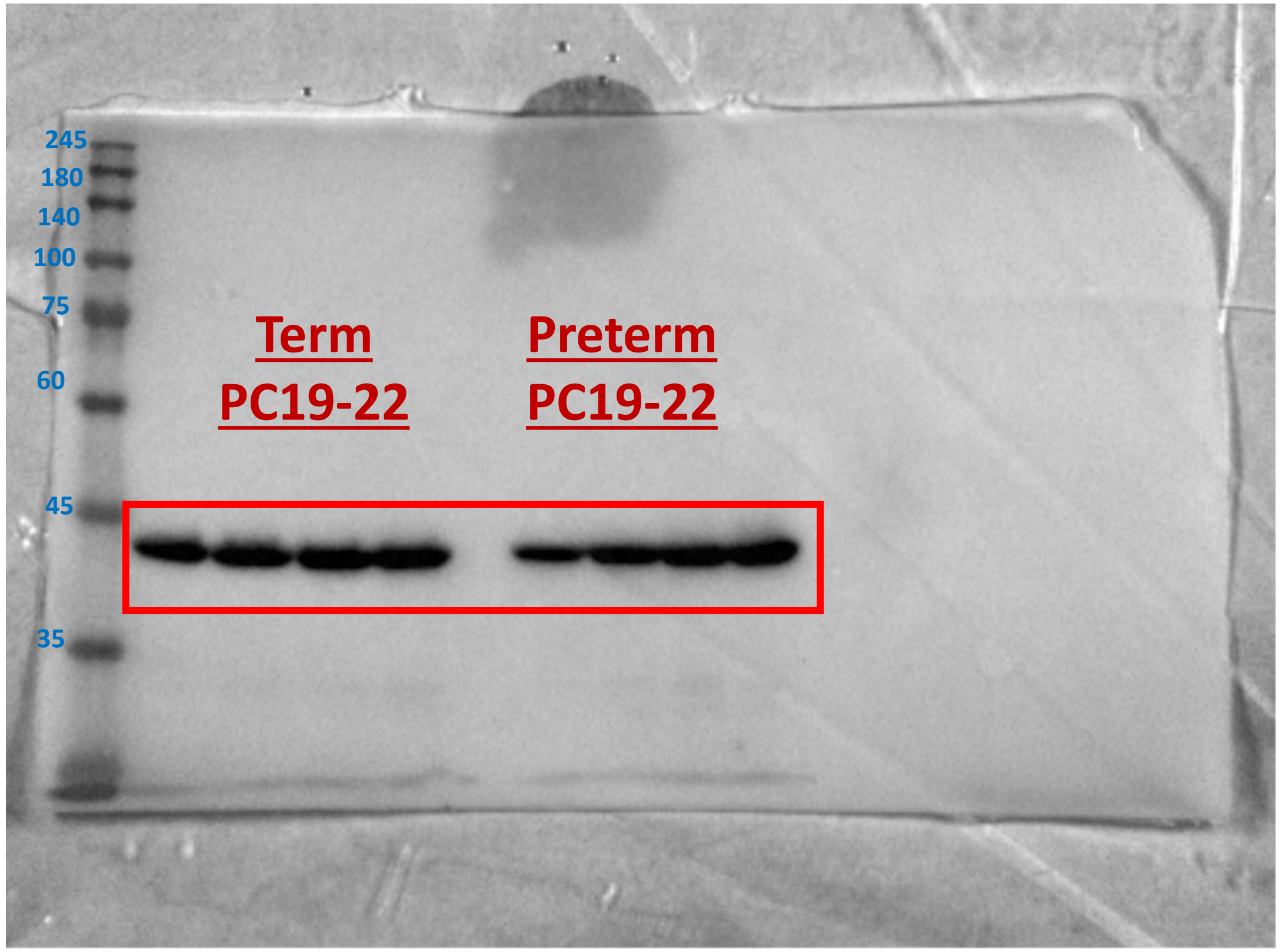

Actin-45 KDa

LHX1 Antibody: Invitrogen-MA5-25920
Ladder: BioGate-BG-PM03
%Gel: 10%

Related to Figure S5- new

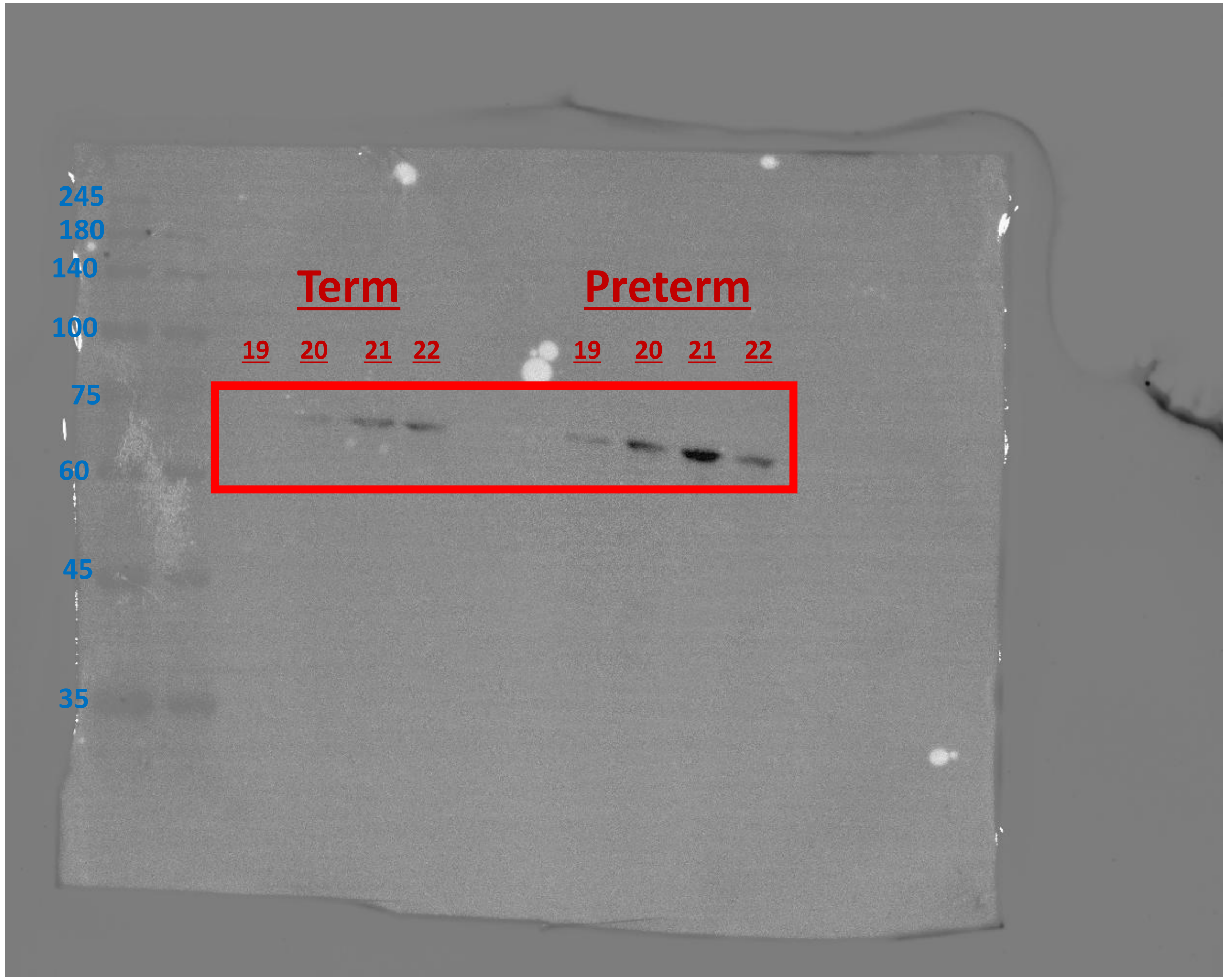

BiP-70-78 KDa

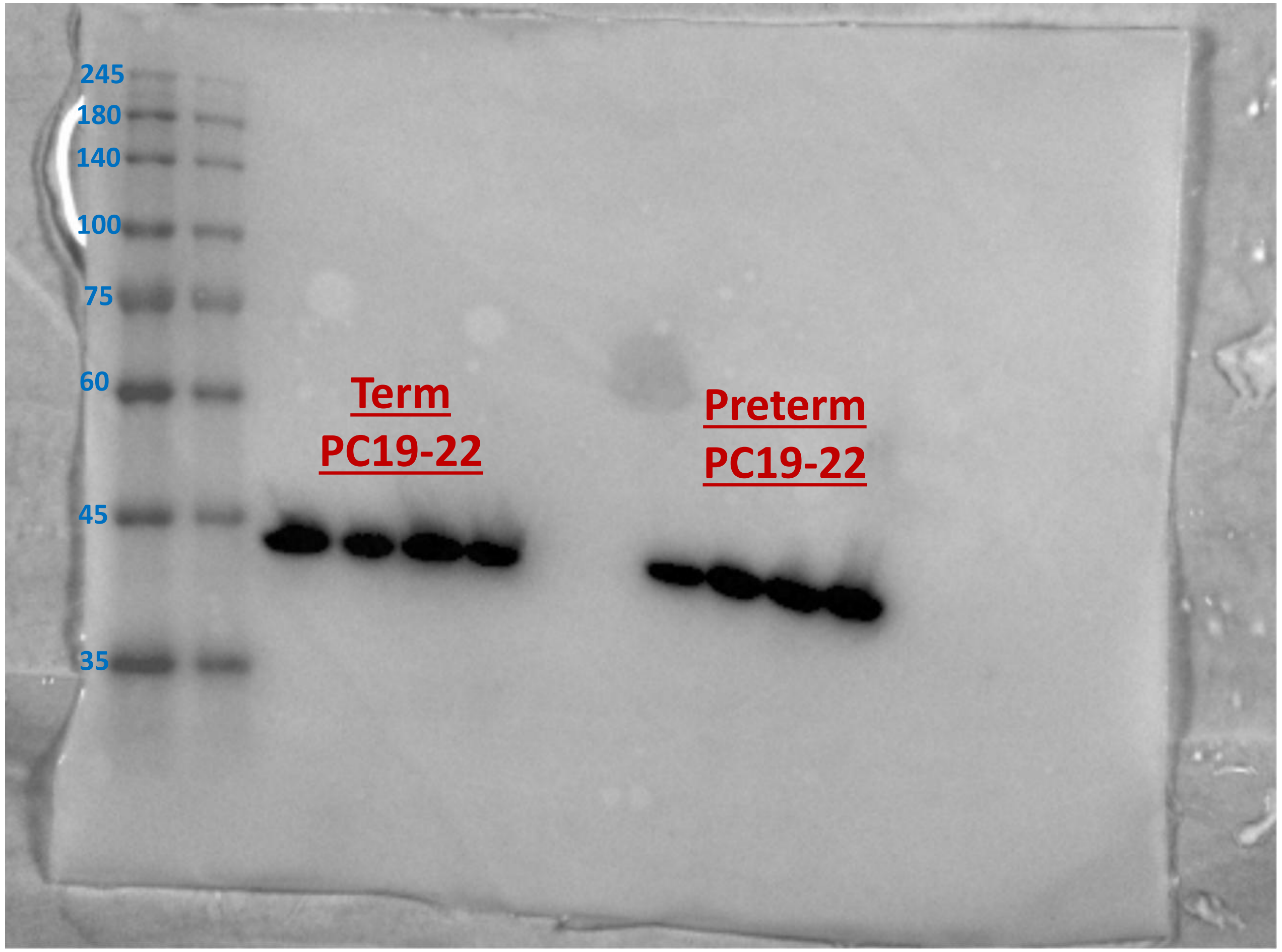

Actin-45 KDa

Ladder: BioGate-BG-PM03

Gel%: 10%

Related to Figure S5- new

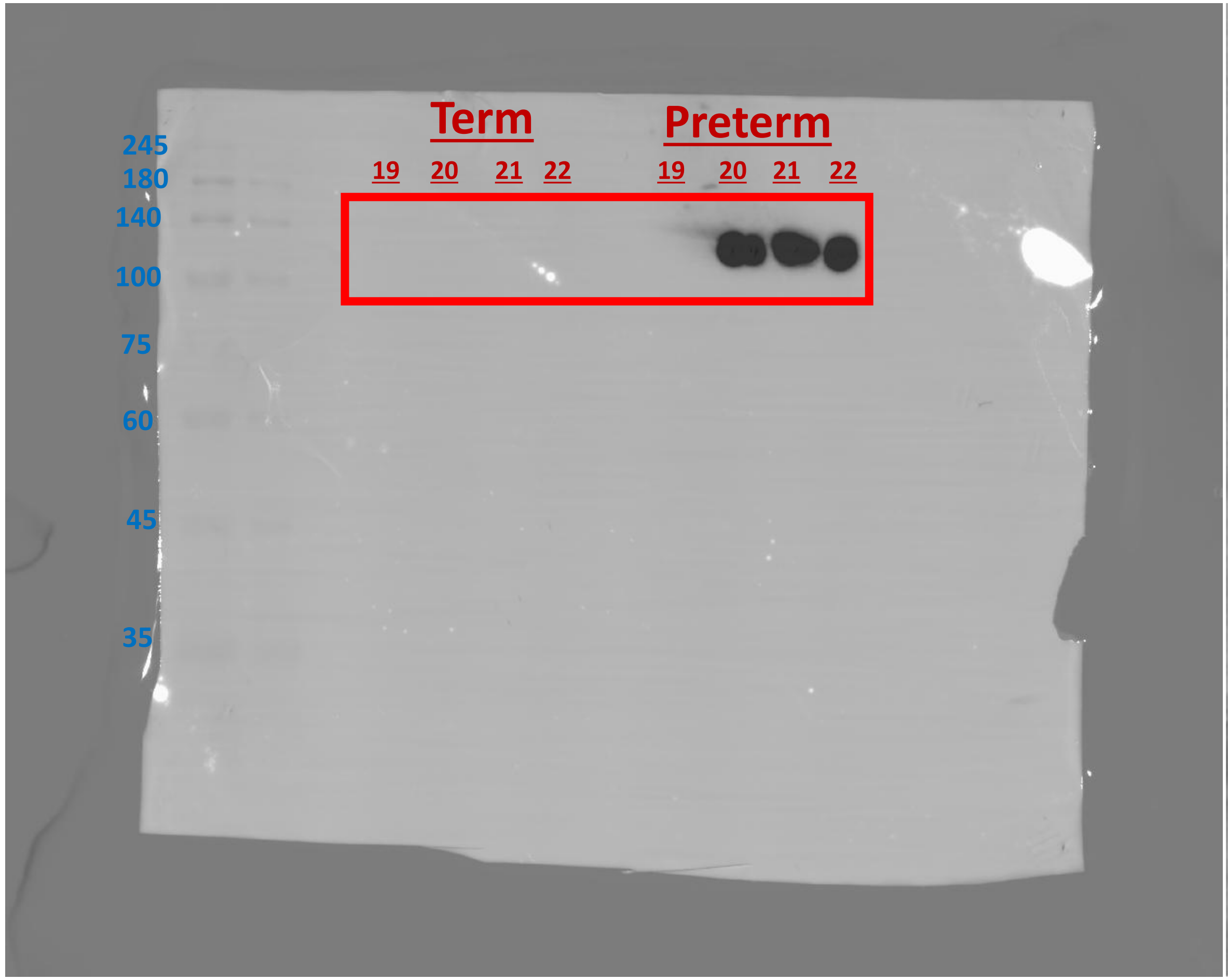

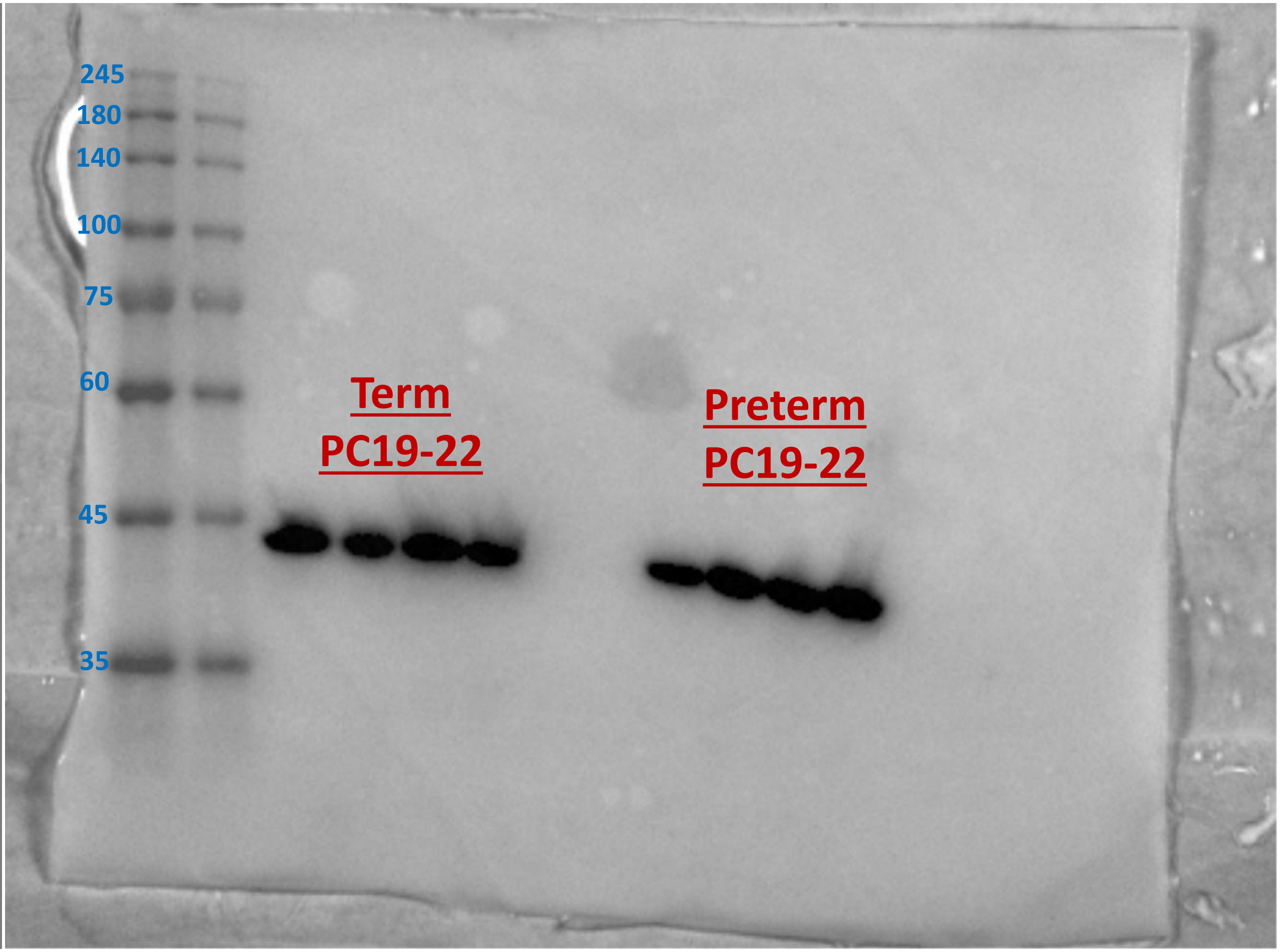

PERK-125-140 KDa

Actin-45 KDa

Ladder: BioGate-BG-PM03

Gel%: 10%
